# Selective, Disruptive Luminescent Ru(II) Polypyridyl Probes of G-Quadruplex

**DOI:** 10.1021/acs.inorgchem.2c03903

**Published:** 2023-01-27

**Authors:** Lorcan Holden, Karmel S. Gkika, Christopher S. Burke, Conor Long, Tia E. Keyes

**Affiliations:** National Centre for Sensor Research, School of Chemical Sciences, Dublin City University, Dublin9, Co. Dublin, Ireland

## Abstract

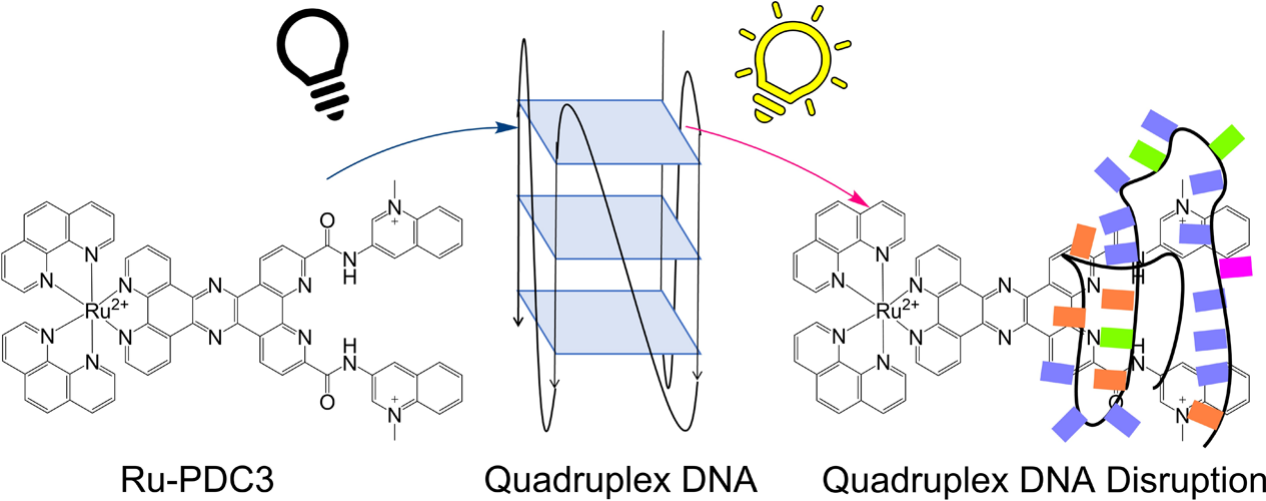

Sensors capable of transducing G-quadruplex DNA binding are important both in solution and for imaging and interrogation in cellulo. Ru(II)-based light switches incorporating dipyridylphenazine (dppz) ligands are effective probes for recognition and imaging of DNA and its polymorphs including G-quadruplex, although selectivity is a limitation. While the majority of Ru(II)-based light switches reported to date, stabilize the quadruplex, imaging/theranostic probes that can disrupt G4s are of potentially enormous value in study and therapy for a range of disease states. We report here, on a Ru(II) complex (Ru-PDC3) that assembles the light switch capability of a Ru(II) dipyridylphenazine complex with the well-known G4-selective ligand Phen-DC3, into a single structure. The complex shows the anticipated light switch effect and strong affinity for G4 structures. Affinity depended on the G4 topology and sequence, but across all structures bar one, it was roughly an order of magnitude greater than for duplex or single-stranded DNA. Moreover, photophysical and Raman spectral data showed clear discrimination between duplex DNA and G4-bound structures offering the prospect of discrimination in imaging as well as in solution. Crucially, unlike the constituent components of the probe, Ru-PDC3 is a powerful G4 disrupter. From circular dichroism (CD), a reduction of ellipticity of the G4 between 70 and 95% was observed depending on topology and in many cases was accompanied by an induced CD signal for the metal complex. The extent of change in ellipticity is amongst the largest reported for small-molecule ligand G4 binding. While a promising G4 probe, without modification, the complex is fully water-soluble and readily permeable to live cells.

## Abstract 2

A supramolecular light switch combining a Ru(II) phenazine unit with a G-quadruplex-selective ligand, Phen-DC3 shows high affinity and specificity for G-quadruplex DNA and disrupts the quadruplex structure on binding.

## Introduction

DNA exhibits a diverse structural polymorphism beyond the double helix in cellulo, including higher-order structures such as triplexes, i-motifs, Y-shaped junctions, and guanine quadruplexes (G4s).^1^ The latter have the propensity to form when guanine-rich sequences are under physiological conditions, where stacked guanine tetrads are assembled through Hoogsteen base pair hydrogen bonding with stacking further stabilized through a central monovalent cation such as potassium, sodium, or lithium.^2^ Although their existence in cells has been well established over the previous two decades through different chemical and biophysical methods, the prevalence and biological role of these structures is still emerging.^3^ G4s feature in crucial steps in DNA translation, oncogene transcription, and telomere stability and are thus cogent sites for both interrogation (imaging) and therapy via their structural stabilization or their destruction.^4^

The evidence for G4 structures in vivo is compelling and has accrued over the past two to three decades from diverse methods including chemical mapping, DNA and RNA stalling, and visualization.^5−11^ Chromatin immunoprecipitation (ChIP) assays with sequencing (ChIP-Seq), using a G4 structure-specific antibody, were performed by Hansel et al., which indicated that there are over 10,000 in the human genome that form G4 DNA structures under physiological conditions.^12,13^ G4s have been found to be particularly prevalent in nucleosome-depleted regulatory regions close to transcription start sites of genes that undergo enhanced transcription. They have also been shown to play a role in cancer, and prevalence is increased in cells showing higher proliferative capacity/immortalization. G4 structures have been demonstrated to play a role in genome instability and are highly prevalent in human oncogenes.^14^ Thus, G4s have emerged as important therapeutic targets.^15^ There is also compelling experimental evidence that G4s exist within chromatin, where they have the capacity to fold from multiple regions of the genome with intrinsic links to genome expression and genome stability.^16−18^ G4s in RNA are less studied than DNA, but computational models and biophysical studies in vitro have provided strong evidence for RNA G4s and indeed in solution RNA quadruplex has been reported to be more thermodynamically stable than DNA analogues.^19^ There is currently less but growing experimental evidence in cellulo to suggest that RNA G4 structures also occur in vivo.^20,21^

The development of small-molecule G4 ligands for therapeutic applications has focused largely on polycyclic planar ligands that can stack on tetrads designed to act as stabilizing agents, such as Phen-DC3, Braco-19, and telomestatin.^22,23^ Stabilizing small molecules can inhibit telomerase activity and induce a DNA damage response (DDR), often providing anticancer activity.^24−29^ Further development of G4 ligands has resulted in additional therapeutic functionalities such as alkylating, oxidizing, and metalating agents.^9,22,30−33^ However, there has been limited development of disruptive G4 ligands that could be used to alleviate the G4-mediated cellular process or manipulate G4s within cells.^34^ Only a small number of G4 ligands have been reported to disrupt G4 structures, and evidence can be challenging due to the complexity of assessing such mechanisms of action.^35−37^ In addition, disruption can be dependent on the G4 type, experimental conditions, and techniques. Nonetheless, G4-disruptive small molecules have significant therapeutic potential, as highlighted recently by Monchaud et al., across diseases with their origins in helicase impairment, including in a number of genetic and neurological disorders.^38^ Design of small molecules that can both probe and disrupt G4 structures offers an elusive but desirable therapeutic prospect.^39^ Indeed, one of the most compelling ways to confirm the G4 presence in vivo is by direct visualization, e.g., through fluorescence imaging. The earliest imaging-based evidence for in cellulo G4 originated from fixed cell studies using labeled antibodies toward G4.^11,21^ G4s have been recently visualized in live cells using fluorescence lifetime imaging (FLIM).^6,40^ Inorganic probes are an attractive option for in cellulo detection of G4s as a number of luminescent coordination compounds are well-established probes of DNA and have been characterized in detail in this regard.^41−49^ Complexes of Ru(II) and Ir(III) have been widely studied as DNA binders with the capacity to report on the structure and also to induce photodamage both in solution and more recently in cellulo.^50−56^ From the imaging perspective, Ru(II) and Os(II) complexes maintain a number of photophysical benefits over organic alternatives including their large Stokes shifts, environmentally sensitive emission, and long-lived phosphorescent lifetimes; combined, these properties make such probes suitable for mutimodal imaging, making them an exciting prospect to explore G4 visualization in live cells.^57−63^

While a number of G4 targeting metal complexes have been shown in solution to exhibit quadruplex binding, their high affinity for duplex DNA is likely to lead to poor discrimination from G4s in vivo, so tuning the structures to improve selectivity is required.^64,65^

Herein, we report on a supramolecular Ru(II) theranostic probe, Ru-PDC3, rationally designed for G4 detection and imaging. Ru-PDC3 exploits two key moieties: [1,10-phenanthroline-2,9-diylbis(carbonylimino)]bis[1-methylquinolinium] (Phen-DC3), one of the families of bisquinolinium dicarboxamide G4 ligands with high affinity and selectivity for quadruplex,^66,67^ and tetrapyrido[3,2-*a*:2′,3′-*c*:3″,2″-*h*:2‴,3‴-*j*]phenazine (tpphz) ligand, one of the families of phenazine containing ligands that instigate the characteristic water light switch effect when coordinated to Ru(II) complexes on DNA binding.^42,68,69^ We demonstrate that the Phen-DC3 affinity and light switch behavior of tpphz are preserved in Ru-PDC3 on G4 binding. We compare the affinity of this complex for different G4 and other DNA structures using luminescence spectroscopy, time-correlated single photon counting (TCSPC), circular dichroism (CD) and Raman spectroscopy, and experimental data, is supported by time-dependent density-functional theory (TD-DFT) calculations. The photophysical data confirm high affinity and specificity Ru-PDC3 for G4 structures compared to helical DNA and that clear discrimination between DNA and G4 binding can be made on the basis of emission lifetime data. Furthermore, marked changes in lifetime and emission intensity with different topologies were observed. In contrast to other reported Ru(II)-based G4 ligands and indeed to Phen-DC3 itself, we have shown using CD titrations that Ru-PDC3 disrupts a variety of G4 structures.^64^

We also report preliminary studies of the uptake and distribution of Ru-PDC3 in live cells and discuss its prospects as a G4 imaging probe.

## Synthesis and Characterization

**Scheme 1:**
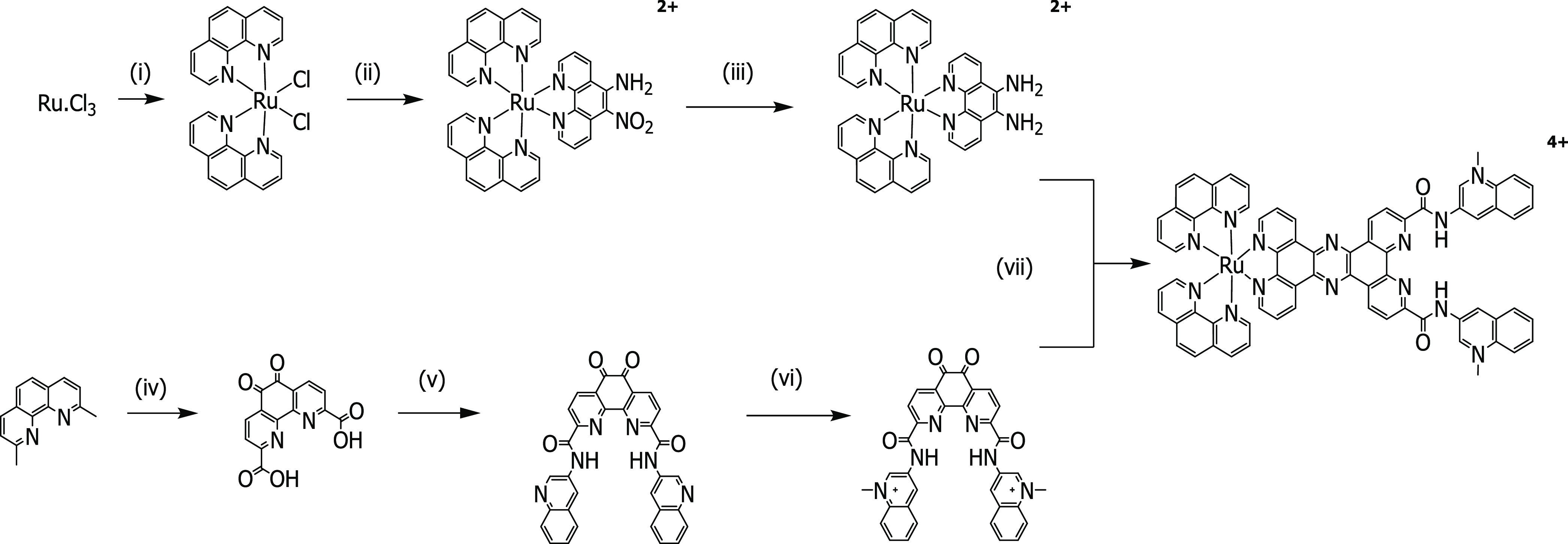
Synthesis of Ru-PDC3 (i) Phenanthroline, LiCl, dimethylformamide (DMF), reflux 8 h; (ii) 5-nitro, 6-amino phenanthroline, EtOH, reflux 5 h; (iii) hydrazine hydrate, Pd/C, EtOH/MeOH, reflux 1 h; (iv) H_2_SO_4_, HNO_3_, KBr, reflux 18 h; (v) 3-aminoquinoline, HATU, *N*,*N*-diisopropylethylamine (DIPEA), DMF, room temperature (rt) h; (vi) methyl-trifluoromethanesulfonate, DCE, reflux 3 h, N2; and (vii) cat. acetic acid, anhydrous MeCN, reflux 72 h.

Synthesis of [Ru(phen)_2_(tpphz-PDC3)]^4+^ (Ru-PDC3) was accomplished according to the strategy outlined in Scheme 1. [Ru(phen)_2_(5,6-diamino-phenanthroline)]^2+^ was obtained through the classic dichloride route as previously reported.^70^ In parallel, a novel Phen-DC3 derivative, phendione-DC3 was synthesized in an analogous manner to that previously reported for G4 targeting phen-DC3; however, the starting material was 1,10-phenanthroline-2,9-dicarboxylic acid-5,6-dione. Conjugation of the acid was completed via 1-[bis(dimethylamino)methylene]-1*H*-1,2,3-triazolo[4,5-*b*]pyridinium 3-oxide hexafluorophosphate (HATU) coupling, and subsequent methylation was achieved by reflux in dichloroethane (DCE) with excess methyl-trifluoromethanesulfonate. To synthesize [Ru(phen)_2_(tpphz-PDC3)]^4+^, a Schiff-base condensation between phendione-DC3 and [Ru(phen)_2_(diamino-phen)]^2+^ was performed in MeCN with a catalytic amount of glacial acetic acid for a 52% yield.

The final product was purified using silica column chromatography and isolated as a PF_6_^–^ salt. Counterion exchange was performed using *tert*-butyl ammonium chloride to generate a water-soluble chloride salt. The excellent water solubility of the complex is an advantage as throughout the spectroscopic and cell-based studies reported herein, there was no requirement for the use of mixed solvent systems to promote solubility. ^1^H NMR and ^13^C NMR were performed to characterize the structure, indicating that a successful condensation reaction occurred and Ru-PDC3 was isolated. Gradient high-performance liquid chromatography (HPLC) was performed to confirm purity by comparing [Ru(phen)_2_(diamino-phen)]^2+^ and Ru-PDC3 chromatograms. HPLC analysis of Ru-PDC3 indicated high purity with no residual [Ru(phen)_2_(diamino-phen)]^2+^ observed (Supporting Information (SI)). Mass spectrometry was performed yielding a *m*/*z* [M]^3+^ = 405.0975 compared with the calculated value of 405.0964, further confirming the successful synthesis of Ru-PDC3.

## Optical and Photophysical Properties of Ru-PDC3

**Figure 1:**
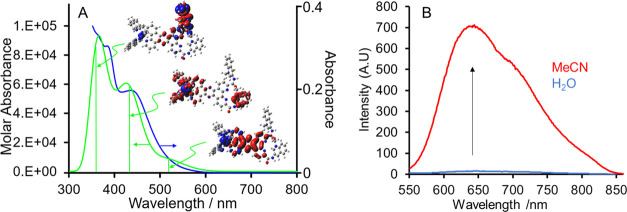
(A) Experimental (blue) absorption of a 10 μM solution of Ru-PDC3 in water and theoretical (green) absorption spectrum along with the electron density difference for the highest oscillator strength transitions modeled at the UB3LYP/LanL2DZ for the excited state. Red volumes are the region of the complex where the electron density is greater in the excited state compared to that in the ground state, and blue volumes are regions where the electron density is depleted in the excited state compared to that in the ground state. (B) Emission spectra of a 10 μM Ru-PDC3 (λ = 450 nm) in DI H_2_O (blue) and MeCN (red).

Representative absorption and emission spectra of Ru-PDC3 are shown in Figure 1A,B The absorption maximum is centered at around 450 nm in water and is red-shifted and the bandwidth decreases slightly in acetonitrile. To gain insight into the absorbance features, TD-DFT calculations on Ru-PDC3 were carried out. The vertical excitation energies and oscillator strengths for the lowest 50 energy states were calculated using the hybrid functional UB3LYP and the LanL2DZ basis set.

Figure 1 overlays the experimental (blue) with computed absorbance spectra (green) of Ru-PDC in water and shows the electron density difference plots for the highest oscillator strength transitions underlying the computed spectrum.

From computation, the absorbance maximum, an ^1^MLCT, contains contributions from two charge-transfer (CT) transitions, originating from the Ru(II) center to the highest occupied molecular orbital (HOMO) phen ligand and to the terminal methylquinolinium on PDC3. The tail of the absorbance contains a lower-energy ^1^MLCT transition, originating from Ru(II) to the tetrapyridophenazine of the PDC3 ligand. Additional absorption features at 393 and 373 nm are associated with MLCT and ILCT transitions between the phenanthroline and the methylquinolinium pendants of the heteroligand.

**Figure 2:**
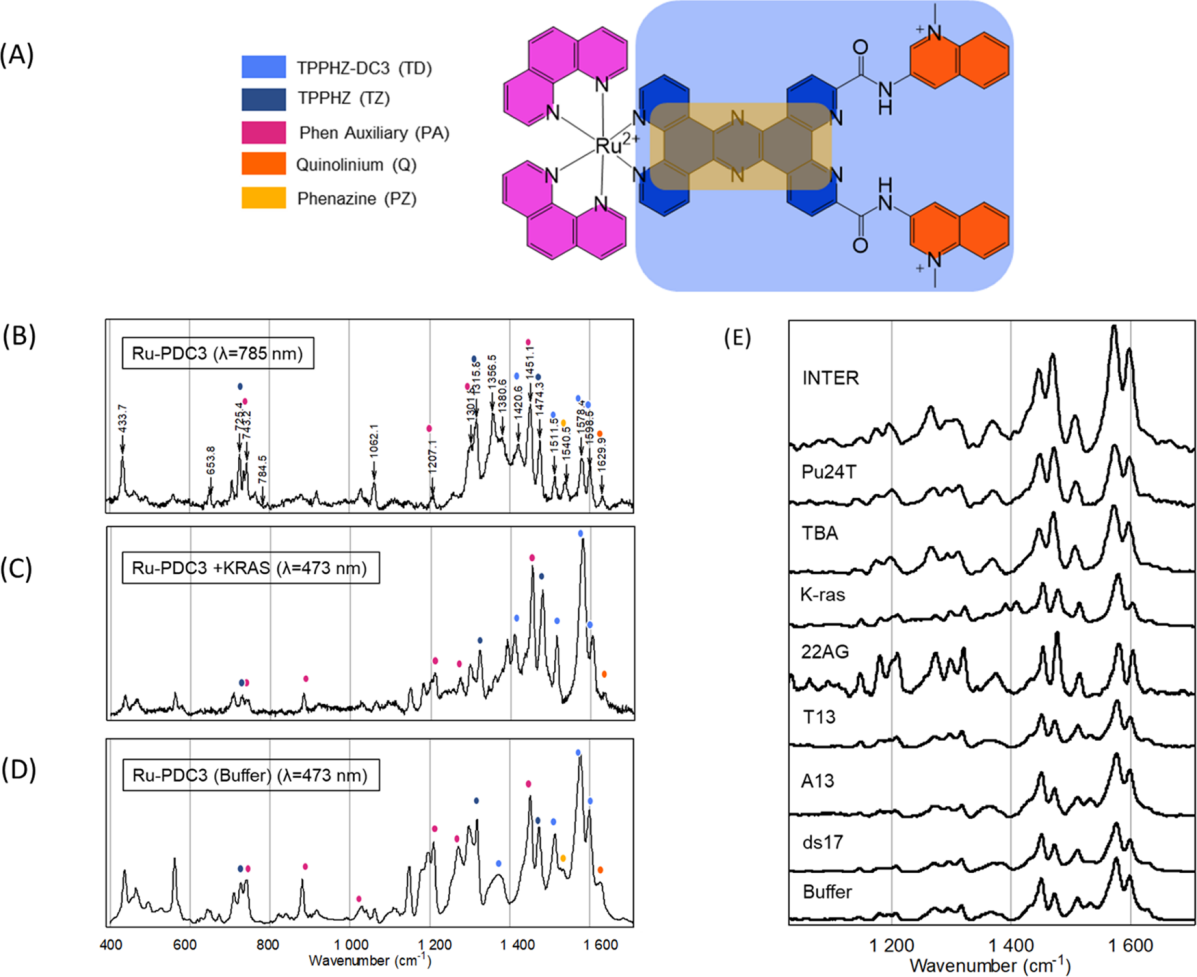
(A) Ru-PDC3 structural assignment for Raman spectroscopy. (B) Raman spectra of Ru-PDC3 (100 μM) (λ = 785 nm). (C) Resonance Raman spectra of Ru-PDC3 (100 μM) bound to K-ras (300 μM) (λ = 473 nm). (D) Resonance Raman spectra of Ru-PDC3 (100 μM) in KPi buffer (10 mM potassium phosphate, 100 mM KCl at pH 7) (λ = 473 nm). (D) Raman spectra of solid-state Ru-PDC3 (λ = 785 nm). (E) Resonance Raman spectra of Ru-PDC3 (100 μM) bound to G4 DNA, ssDNA, and double-stranded DNA (300, 500, and 500 μM, respectively) in KPi buffer (λ = 473 nm). Spectra are normalized to the vibrational band at 1508 cm^–1^.

We compared the Raman spectrum (Figure 2D) of Ru-PDC3 obtained under a 785 nm laser excitation with the resonantly excited Raman spectrum (λ = 473 nm) (Figure 2B) to gain a deeper understanding of the nature of the optical transitions. And, to aid the interpretation of the Raman spectrum, we compared the Raman spectrum under nonresonance (785 nm) excitation conditions with the theoretically predicted Raman spectrum. These spectra are shown in SI materials. There is excellent agreement between the experimental and predicted Raman spectra of Ru-PDC3; the table in the SI provides the frequencies and assignments of the vibrational bands. Based on this interpretation, the vibrational modes are associated primarily with three ligand moieties of the complex: the phen ligands, the tpphz ligand, and the terminal methylquinolinium termini are identified and, for simplicity, are indicated as pink, blue, and orange in the color-coded scheme in Figure 2A and marked on the resonance Raman (RR) spectra shown in Figure 2B–D.

Resonance Raman (RR) spectroscopy was carried out under a 473 nm excitation, which is close to the λ_max_ of the complex in water. Under such conditions, the Raman active vibrational modes associated with the chromophores participating in the electronic transition undergo a dramatic enhancement in their vibrationally induced polarizability and thus in their Raman scattering cross section. This effect leads usually to between 5 and 7 orders of magnitude enhancement in Raman scattering from vibrational modes associated with the Frank–Condon relaxation and so can provide clear insight into the origin of the optical transition.

The Raman spectrum of Ru-PDC3 at a 473 nm excitation shows notable complexity for a resonantly enhanced spectrum. All of the vibrational modes observed under nonresonant excitation are also observed in the resonantly enhanced spectrum, albeit with a markedly different intensity profile. Vibrations associated with all three moieties, phen, tpphz, and the more remote methylquinolinium unit (CC stretching mode at 1630 cm^–1^), are evident in the RR spectrum. This observation is consistent with the computed assignment of the electronic spectrum, which indicates that the lowest two energy optical transitions, both of which are expected to contribute at 473 nm, are complex MLCT transitions that extend across all three ligand moieties.

Figure 1B shows that Ru-PDC3 exhibits light switch properties consistent with the behavior reported previously for related tpphz- and dipyridophenazine (dppz)-coordinated Ru(II) complexes. In aqueous media, the emission is essentially extinguished. In aprotic environments, in contrast, as shown here in acetonitrile, intense emission from the ^3^MLCT state is observed with a maximum at 645 nm, which shows some vibrational structure with a shoulder evident at around 700 nm, extending to 850 nm. The emission decay of Ru-PDC3 in acetonitrile is shown in the SI. Exciting at 470 nm, the decay conforms to biexponential kinetics: with τ_1_ of 223 ± 2 ns (intensity amplitude of 15%) and τ_2_ 82 ± 1 ns (intensity amplitude of 75%) under aerated conditions. On deaeration, we obtain τ_1_ of 351 ± 7 ns (intensity amplitude of 9%) and τ_2_ of 95 ± 3 ns (intensity amplitude of 91%), i.e., only the long-lived component shows significant oxygen sensitivity, but the amplitude of the short-lived component increases. Impurity can be excluded, as beyond the confirmation of purity from HPLC and NMR, as there is no batch-to-batch variation in the amplitude of the decays across different syntheses. The biexponential nature of the decay for Ru(II) polypyridyl complexes is concentration-independent, so not attributable to aggregation, and may be due to genuine dual emission, as TD-DFT calculations indicate that there are orbitals on the dppz ligand with energies close to the lowest unoccupied molecular orbital (LUMO) that extend over the PDC ligand. Alternatively, it may originate from photoinduced electron transfer between the ^3^MLCT and the quinolinium moieties. Although the latter is expected to have a reduction potential in the region of −1 eV, that when applied to the Rehm–Weller equation, and assuming a Ru(II)/(III) oxidation potential of 1.3 eV and 610 nm emission maxima at 77 K, yields a negligible driving force for such a process.

## DNA Binding Affinity and Selectivity

To investigate the affinity and selectivity of Ru-PDC3 for G-quadruplex DNA, its interaction with duplex, calf thymus (ct) DNA, single-stranded (ss) DNA, and a range of human G-quadruplex forming oligonucleotides of representative topology were evaluated and compared using emission intensity, lifetime studies, and circular dichroism.

**Figure 3:**
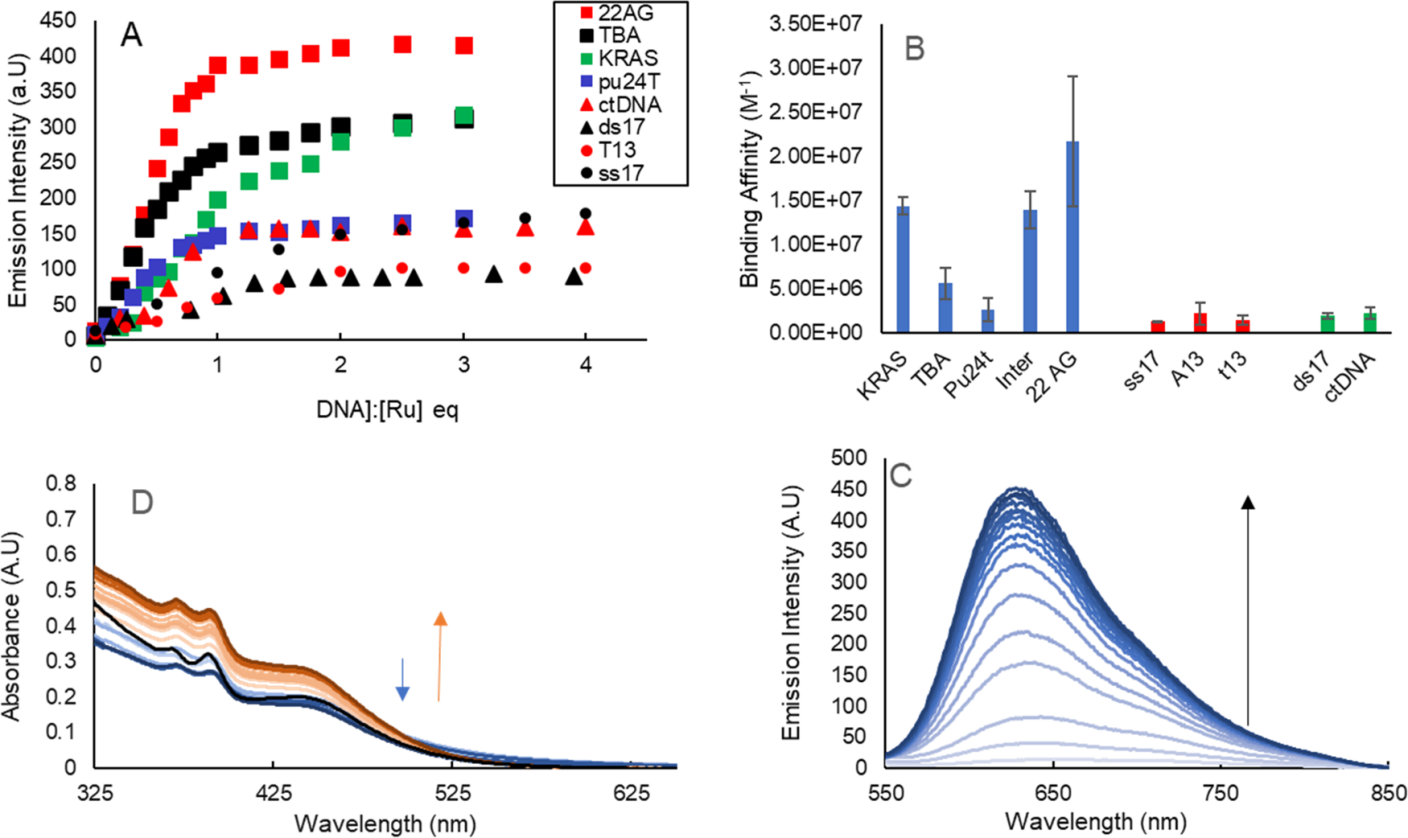
(A) Representative titration binding curves for Ru-PDC3 against a series of DNA structures (ds17, T13, 22AG, TBA, and K-ras) Concentration is calculated as a strand equivalent for G4s and base pair equivalents for dsDNA base equivalents for ssDNA. (B) Calculated binding affinities based on binding curves for G4s (blue), ssDNA (red), and dsDNA (green). (C) Representative emission titration for Ru-PDC3 against 22AG. (D) UV–vis absorption of Ru-PDC3 when titrated against 22AG.

First, emission titrations were performed to assess the binding affinity of Ru-PDC3 against the panel of nucleic acid structures. The titration of nucleic acid against the complex resulted in emission switch-on and evolution of luminescence intensity with increasing nucleic acid concentration, across all of the nucleic acid materials explored. A representative example showing titration against 22AG is shown in Figure 3C. However, the extent of emission increases and the band shape varied significantly across ds, ss, and G4 DNA (see SI materials).

The binding affinities of Ru-PDC3 were determined by fitting luminescence titration curves to the model described in the SI, and the data is tabulated in Table S2 (SI) along with the representative fitted plots. (Of note, attempts to study the affinity of the free ligand PDC3 with that of the complex were hampered by the poor aqueous solubility of the ligand.) Representative fluorescence binding data across all of the nucleic acid materials is graphed in Figure 3A. Interestingly, binding affinities for dsDNA and ssDNA were comparable and similar to values previously reported for Ru-dppz complexes in buffers of high salt concentration (>50 mM NaCl). Affinities for ds17 and ctDNA were determined as (1.9 ± 0.3) × 10^6^ and (2.3 ± 0.6) × 10^6^ M^–1^, respectively. For single-stranded DNA binding affinities for ss17, T13 and A13 were determined as (1.3 ± 0.4) × 10^6^, (1.4 ± 0.5) × 10^6^, and (2.2 ± 1.3) × 10^6^ M^–1^, respectively; these values are markedly high and comparable to affinity with dsDNA and may be attributed to the 4+ positive charge on the complex.^71,72^

**Figure 4:**
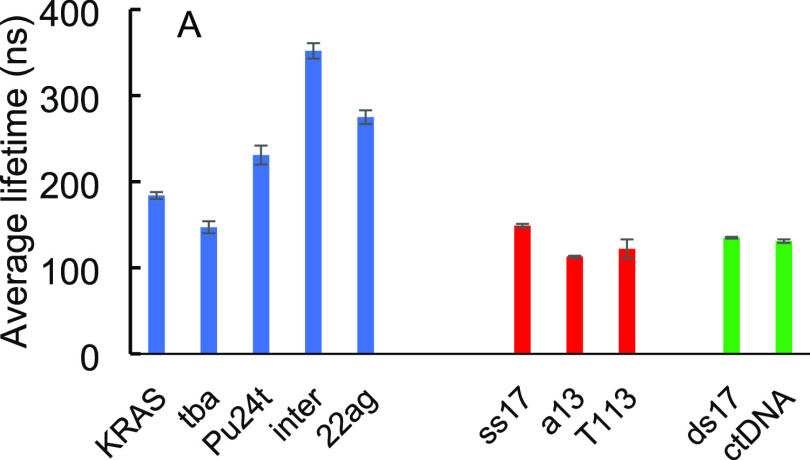
Intensity-weighted average lifetimes (ns) of Ru-PDC3 upon saturation binding to a panel of DNA structures. Average lifetimes are an average of three repeats and obtained using the PicoQuant Fluofit software.

For G4 sequences (Table S1), the quadruplexes were annealed as a concentrated stock in K^+^ buffer. K-ras and Pu24T are parallel G4s, TBA is an antiparallel G4, 22AG is a mixed hybrid structure when annealed in a K^+^ environment, and INTER is a four-stranded intermolecular G4. Ru-PDC3 binding affinity for K-ras, INTER, and 22AG was at least an order of magnitude greater than recorded for ds or ssDNA. The affinities were determined as (1.4 ± 0.1) × 10^7^, (1.4 ± 0.2) × 10^7^, and (2.2 ± 0.7) × 10^7^ M^–1^, respectively, for K-ras, INTER, and 22AG quadruplexes. The relative emissivity of the bound complex is reflected in the intensity of the graphed plots in Figure 3A as the excitation conditions are identical in each case and followed the following pattern ss < ds < G4, with a notably dramatic increase in emissivity on binding to INTER where emission intensity was roughly an order of magnitude higher than for other G4s and weak emission enhancement, comparable to ss and dsDNA for Pu24T.

The bar chart in Figure 3B shows a clear discrimination in the affinity of Ru-PDC3 for G4 structures over dsDNA and ssDNA. Notably, Ru-PDC3 shows the highest affinity for the mixed hybrid quadruplex 22AG with a notably lower affinity toward TBA and particularly Pu24T quadruplexes, where affinity was (5.6 ± 1.7) × 10^6^ and (2.6 ± 0.1) × 10^6^ M^–1^, though still greater than duplex DNA.

The significant variance in binding affinities of Ru-PDC3 for different G4s is interesting and may be explained by considering that binding sites for G4s will be different, depending on the topology and loop structure. The difference in the binding affinity between Pu24T and K-ras is interesting, as they both (confirmed from CD spectroscopy, vide infra) are expected to have parallel structures. However, while their strands are oriented in the same direction and both contain three quartets, their loop structures are distinctive. K-ras exhibits three regular propeller loops but Pu24T, one of seven nuclease-hypersensitive elements (NHEs), NHE III1 c-myc gene, has an unusual fold-back and reversal loop that bridges across the tetrad layers, which are likely the origin of the difference in binding affinity. Differences in affinity between organic ligands between parallel structures have been noted previously.^73−75^ The increased affinity observed for Ru-PDC3 when compared to other reported Ru(II) analogues may, in part, be associated with the additional electrostatic interactions as a result of the positively charged quaternary nitrogens, for example Su et al., Δ- and Λ[Ru(phen)_2_(dppz)]^2+^ were reported to bind to 22AG with an affinity of 4.6 × 10^6^ and 2.7 × 10^6^ M^–1^.^64^ Further reports by Ji et al. indicated a binding affinity of [Ru(bpy)_2_(dppz)]^2+^ to 22AG to be 1.4 × 10^6^ M^–1^.^76^ While the additional charge of Ru-PDC3 may increase the binding affinity with G4s, we hypothesize that binding to other non-G4 DNA structures is less specific as a result of steric hindrance from the bisquinolinium functional groups. The differences observed in enhancment of fluorescence intensity/quantum yield on saturation binding we attribute to differences in the binding mode resulting in different degrees of phenazine exposure to the aqueous environment. Figure S21 illustrates the greater emissivity of Ru-PDC3 when bound to the INTER quadruplex in comparison to other G4s indicating effective shielding of Ru-PDC3, suggesting different binding modes. Interestingly, there does not appear to be a correlation between emission intensity and binding affinity between G4s. The binding mode to G4s is likely dependent on the sequence and folding, with past studies on ruthenium polypyridyl complexes binding to G4s suggesting binding through loops, bulges, and intercalation.^77−79^

## Interaction of Ru-PDC3 with DNA

Although there is clear discrimination in terms of emissivity and affinity of Ru-PDC3 between the DNA structures and individual quadruplexes, as binding stimulates the light switch effect across all nucleic acid structures explored, structural discrimination will not be possible in conventional fluorescence imaging. Fluorescence lifetime imaging (FLIM), however, can provide discrimination if there is a sufficient distinction between the lifetimes of the complex as it associates with different structures. Vilar et al., for example, have reported recent success in discriminating the response of organic dyes against G4s and dsDNA in FLIM.^6,40,80^ To investigate the prospect of discriminating DNA secondary structures on the basis of lifetime, we carried out TCSPC on the Ru-PDC3 with each of the DNA structures. Measurements were performed under binding conditions with a [DNA]/[Ru] ratio of [3]:[1] for quadruplexes, [5]:[1] for dsDNA, and [5]:[1] for ssDNA. All DNA measurements were performed in a buffer of 10 mM potassium phosphate and 100 mM KCl at pH 7 under aerated conditions.

Table 1 presents the resulting lifetime data. As described, the complex emits with biexponential decay kinetics in MeCN while emission is extinguished in aqueous media. In the presence of nucleic acid, emission switches on and decays according to triexponential decay kinetics. τ_1_ is >150 ns and τ_2_ is >30 ns. With the exception of A13 and T13 ssDNA, the behavior is comparable to those observed for the parent complex in acetonitrile. In addition, a short-lived component, τ_3_, is observed with values of <40 ns and <10 ns in the case of A13 and T13 ssDNA. This component was not observed in MeCN and is tentatively attributed to emission from complex bound to DNA but in an aqueous-exposed environment. Comparable kinetics have been noted previously for related dppz complexes of Ru(II) in association with DNA structures including quadruplex.^77^ The long-lived components of the decay, τ_1_ and τ_2_, are attributed to the dual exponential decay observed in acetonitrile, originating from the complex in a binding mode that offers the best protection from water. τ_3_ is tentatively attributed to the complex in a binding configuration that leaves the complex more exposed to aqueous solution as on the basis of the electrochemistry and optical properties of the complex we do not expect direct electron transfer to the DNA structures. Notably, there is a clear discrimination in the lifetime on binding to ds and ssDNA structures and the quadruplexes. This is presumably attributed to different modes between, e.g., duplex and quadruplex. Ruthenium complexes have been reported to intercalate between guanine tetrads in intermolecular G4 structures in addition to terminal capping reported for mononuclear and binuclear complexes.^46,79^ τ_1_, in particular, is sensitive to quadruplex binding with decays in the range of 389–430 ns, which is roughly 100 ns longer in most cases compared to τ_1_ when bound to ds or ssDNA. Indeed, the lifetime of this component exceeds the lifetime observed in deaerated acetonitrile, indicating, in all cases, that at least one mode of quadruplex binding offers significant protection to Ru-PDC3 from the aqueous environment. However, notably, the amplitude of τ_1_ varies significantly across nucleic acids.

**Table 1:** Lifetime and Amplitude of Each Component Obtained from TCSPC Measurements (λ = 450 nm)^a^

	τ_1_ (ns)	α1 (%)	τ_2_ (ns)	α2 (%)	τ_3_ (ns)	α3 (%)	τav.1 (ns) intensity
aerated (MeCN)	223 (±2)	14.3	82 (±1)	75.7			130 (±4)
deaerated (MeCN)	351 (±7)	8.9	95 (±3)	91.1			163 (±3)
K-ras	389 (±6)	8.5	93 (±5)	44.0	24 (±6)	47.5	184 (±4)
22AG	426 (±8)	14.5	110 (±5)	39.2	19 (±3)	46.4	275 (±8)
Pu24t	430 (±30)	8.7	101 (±10)	37.3	19 (±4)	54.0	231(±11)
INTER G4	418 (±13)	43.6	197 (±9)	40.4	13 (±2)	16.0	352 (±9)
TBA	395 (±17)	4.5	90 (±8)	44.7	37 (±4)	50.8	147 (±7)
ds17	284 (±5)	31.7	74 (±1)	57.0	16 (±1)	11.3	135 (±1)
ctDNA	291 (±4)	6.5	62 (±1)	53.4	12 (±1)	40.1	131 (±2)
ss17	329 (±12)	6.6	75 (±1)	43.4	17 (±1)	50.0	149 (±2)
A13	152 (±4)	8.4	40 (±2)	52.4	4 (±1)	39.2	113 (±1)
T13	205 (±26)	9.8	46 (±5)	51.3	8 (±2)	37.8	122 (±11)

a Ru-PDC3 (10 μM) in the presence of G4s (30 μM), dsDNA (50 μM), and ssDNA (50 μM). Values are the average of three repeats. Triexponential fit was applied using the PicoQuant Fluofit tail-fit analysis software.

Consistent with the steady-state fluorescence, binding to 22AG yields one of the longest τ_1_ emission decays from the ruthenium center at 426 (±8) ns, indicating that at least one mode of binding to this G4 provides significant protection of the tpphz center. Although overall τ_1_ is fairly consistent across all of the quadruplex topologies, ranging from approximately 390 to 430 ns, there is no correlation between affinity for the G4s and τ_1_. For example, τ_1_ on TBA association is comparable to 22AG at 426 (±8) ns even though the affinity was dramatically lower for this polymorph. Rather, it is taken to reflect the most phenazine-protected binding mode for a given topology. The amplitudes of the decay components τ_1_ and τ_3_ are taken to reflect the relative predominance of at least two binding modes. For example, for Ru-PDC3 associated with INTER G4, over 40% of the intensity amplitude of the decay comes from the long-lived component (418 ns), indicating that a binding mode that protects the phenazine is more prevalent in this complex, compared, for example, to K-ras or TBA where 4 and 8% of the amplitude of decay are derived from the long-lived component. Indeed, in all G4s, τ_3_ dominates the decay, at nearly 50% of amplitude. τ_2_ is relatively stable both in value and in amplitude across G4s, with the exception of INTER G4 where it is roughly 50% of the decay amplitude. Interestingly, dsDNA, although showing a relatively short τ_1_ compared to G4 (and as expected similar to ctDNA), has a significant amplitude of >30% for this component of the decay, consistent with intercalation. The notable variation of lifetime and relative amplitudes of the contributing components may provide the possibilities to distinguish between different G4s in a cellular environment, but the complexity of the decays may make it difficult to analyze in practice, where average lifetimes are easier to collect in imaging. Indeed, discrimination remains clear when, as shown Figure 4, the averaged lifetimes are compared as the shorter-lived components contribute greater amplitude in the lower affinity polymorphs. 22AG and INTER show clear discrimination from the other polymorphs from both intensity and amplitude-averaged lifetimes. Overall, the lifetime data shows promise, from the perspective of FLIM, that DNA forms maybe discriminated on the basis of lifetime; an increase in intensity-weighted lifetimes across all quadruplex environments is clear compared to that of dsDNA and ssDNA environments.

## Influence of Ru-PDC3 on the DNA Structure

As G-quadruplex DNA is optically active, circular dichroism (CD) spectroscopy is a useful method to interrogate structural changes to G4s induced by their interaction with Ru-PDC3. The G4 topologies for each sequence used here can be readily distinguished by CD spectroscopy. The metal complex used herein is a racemic mixture so as expected does not show optical activity in the absence of DNA. In all cases, the CD of the G4 in the K^+^ buffer was consistent with the expected topography, as reported previously.^81−85^ Peak maxima and minima were observed at approximately 264 and 245 nm, respectively, for K-ras and Pu24T, consistent with their parallel topology, at 295 and 260 nm, respectively, for TBA consistent with the antiparallel structure, and at 295 max, ≈260 max, and 245 nm, respectively, for 22AG, which has a mixed hybrid structure.^86^ Peak maxima for INTER were observed at 215 and 260 nm consistent with a four-stranded intermolecular quadruplex. The peak maxima or minima shifts, changes of amplitude, or loss of these features on G4 interaction with small molecules can reflect the formation or disruption of the G4 structure.

In these experiments, Ru-PDC3 was titrated into a solution where G4 DNA was at a constant concentration to follow changes to the G4 topology. Recently, such an assay has been established to investigate known stabilizers and disruptors, where it was shown that a reduction in the CD peak intensity at a characteristic quadruplex wavelength can relate to the disruption of G4s.^38^

**Figure 5:**
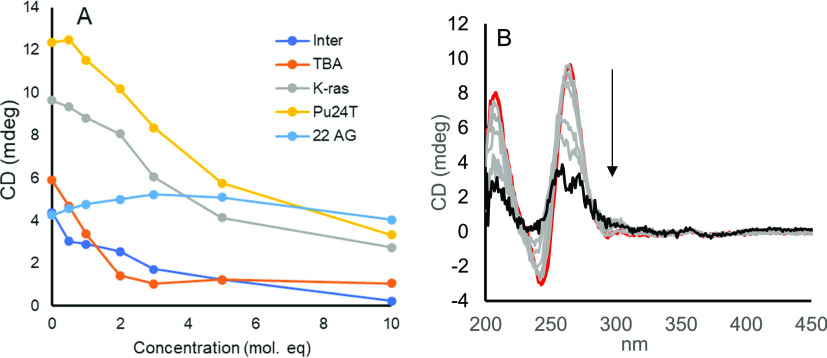
(A) Intensity of the peak CD signal from different G4s as a function of Ru-concentration. Ru-PDC3 concentration ranges from 0 to 10 mol equiv to [Ru]/[G4]. Quadruplex concentration is maintained at 5 μM in KPi buffer. CD signal intensity was monitored at peak maxima that depended on the topology, which were 265 nm for Pu24T and K-ras, 295 nm for 22AG and TBA, and 210 nm for INTER. (B) Representative CD spectra of K-ras (5 μM) when titrated against increasing equivalents of Ru-PDC3 (0–10 mol equiv of [Ru]/[G4].

Remarkably, as illustrated in Figure 5, the titration of Ru-PDC3 against the above-described G4s resulted in exceptional disruption of the G4 structure. This was evident as a significant loss of ellipticity with the addition of the metal complex. Such a disruptive response is relatively unusual upon small ligand binding, particularly so for ruthenium polypyridyl complex binding, where mainly stabilization of quadruplexes has been reported to date. One exception was a mononuclear complex of [Ru(phen)_2_(PHEHAT)]^2+^ where similar reductions in the ellipticity of Tel22 G4 at high ratios (> 5:1 ratio of Ru/G4) were evident.^78^

When titrated against the antiparallel TBA G4, the signal at 295 nm was reduced by 82% at 5 equiv of Ru. Such a dramatic, near-total loss of ellipticity is exceptional for a small molecule, particularly under such a low [Ru]/[G4] ratio and under high salt conditions (100 mM KCl).

Notably across several G4 topologies, induced CD (ICD) was observed in features associated with the metal complex. Following the titration of 3 equiv of Ru(II) against TBA, ICD is observed at 400 and 260 nm that grows upon further titration. Such a response may indicate the selective affinity of one enantiomer, Δ or Λ, for the G4 structure. Similarly, the CD signal of the two parallel complexes Pu24T and K-ras was greatly reduced when titrated against Ru-PDC3; both G4s exhibited remarkably similar destabilization with a total reduction in ellipticity at 265 nm of 71 and 73% for Pu24T and K-ras, respectively. As the destabilization is similar for both parallel G4s, it is possible that the similarity relates to the influence of the sequence direction and loop structure. Unlike TBA, ICD signals did not emerge to a significant extent during the titration. Titration of Ru-PDC3 against the INTER G4 resulted in the most extensive disruption of the quadruplex signal, and these changes were also accompanied by an intense ICD. Ellipticity decreases by 95% at a 10:1 [Ru]/[G4] ratio at 210 nm. The intense ICD of Ru-PDC3 occurring 248 and 264 nm obscures potential changes in ellipticity arising from the G4 topology, to overcome this analysis was performed at multiple wavelengths. Interestingly, Ru-PDC3 did not induce complete disruption of the mixed hybrid quadruplex 22AG. The decrease in ellipticity at 265 nm and a positive ellipticity at 295 and 245 nm indicate rather, a conformational change to an antiparallel quadruplex in which guanines are stacked in alternating orientations. This ligand-induced change is remarkably similar to the previously reported interaction between Phen-DC3 and a human telomeric G4 analogous to 22AG, where a K^+^ ion was ejected by Phen-DC3 followed by the stabilization of a two-quartet antiparallel quadruplex.^87,88^

The promising disruptive activity of Ru-PDC3 was unexpected and, we believe, currently unparalleled in small-molecule G-quadruplex association across the literature. As a comparison, Tmpy4, one of the best-known G4 small-molecule ligands, results in less disruption across a narrower range of G4s. As reported by Mitteaux et al., when Tmpy4 is titrated against 22AG in K^+^ buffer at a ratio of [10]:[1] ligand to G4, a reduction in CD ellipticity of 68.7% was observed when monitored at 293 nm.^38^

### Resonance Raman Spectroscopy of G4-Bound Complex

The resonance Raman spectroscopy of the Ru-PDC3 was examined under a 473 nm excitation following its incubation with each of the nucleic acids studied. The absorbance spectra, Figure 2E, showed that on DNA association, hyperchromicity of the Ru-PDC3 absorbance spectrum was observed but the spectral features remained unchanged, so we expect that resonance remains approximately the same. Under resonance conditions, only the Raman features of Ru-PDC3 are evident in the spectrum. Association with the nucleic acids exerted a subtle but significant influence on the resonance Raman spectrum. There were small shifts in the key Raman features on nucleic acid association, and these were greater for G4 than for DNA, but, in particular, there are clear changes in relative intensities of the phen-based vibrational modes between 1400 and 1500 cm^–1^ that increase in relative intensity compared to the tpphz features that dominate above 1500 cm^–1^. Similar behavior has been noted previously in dppz-based Ru(II) complexes on DNA binding. In addition, most notably, the vibrational mode at 1534 cm^–1^ isolated on the phenazine is lost, exclusively on quadruplex binding.^89^ The feature although reduced in intensity on duplex binding remains evident on association with all other nucleic acid materials, especially single-stranded structures. This is a potentially important finding that may permit the discrimination of G4 structures in resonance Raman in cells.

## Live Cell Uptake and Cytotoxicity

Ultimately, for the application of these complexes as imaging probes or in therapy, cell permeability and targeting are important. Given the high cationic charge of Ru-PDC3 and its water solubility, we explored whether the complex, without further modification, is permeable to live cells. We examined uptake by A549 lung carcinoma cells using confocal fluorescence microscopy. The water-soluble complex was introduced to cells over a range of concentrations (25–100 μM) in the absence of light, from an aqueous buffer without the inclusion of an organic solvent for dissolution. Under these conditions, the complex exhibited good uptake in live cells and the resulting intracellular distribution was observed to be consistent across this concentration range. The optimized concentration for imaging was determined to be 50 μM based on the balance between image brightness and cytotoxicity, discussed vide infra.

**Figure 6:**
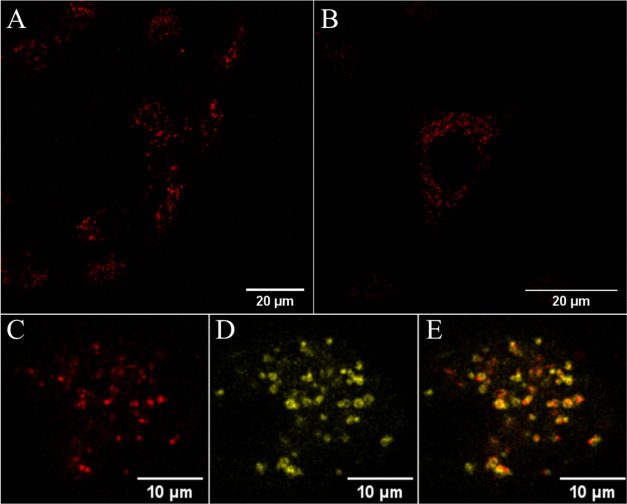
Confocal images showing the uptake of (A, B) Ru-PDC3 in live A549 cells at 50 μM following a 24 h incubation in the absence of light. A 475 nm white light laser was used to excite the ruthenium complex, and emission was collected between 550 and 800 nm. In colocalization studies, cells were incubated with (C) 50 μM of Ru-PDC3 for 24 h and costained with (D) Rab7a-GFP that indicated confinement in some late endosomes evident by the overlap of the two channels in the (E) overlay image (Pearson’s coefficient, *r* = 0.73 ± 0.03). Rab7a-GFP was excited at 488 nm, and emission was collected between 490 and 540 nm.

Figure 6A shows representative cell imaging following the incubation of Ru-PDC_3_ at 50 μM in A549 lung cancer cells for 24 h. Due to the light switch properties of the complex in the aqueous environment, monitoring its uptake and true distribution within the cell is challenging as it emits only from aqueous exclusive environments. Nonetheless, following uptake, the complex exhibits a bright punctuate emission from within the cytoplasm, although it appears to be largely nuclear excluding (Figure S41). The marked intensity of the emission indicate that it is associated with either membranous structures or nucleic acid materials, and the punctate distribution indicates that it is emitting from within organelles or endosomes.

The negative transmembrane potential of mammalian cells generally favors the uptake of positively charged complexes via energy-independent mechanisms such as passive diffusion, although the punctate distribution of the complex may suggest endosomal entrapment. Therefore, to examine the uptake mechanism, A549 cells were pretreated with oligomycin and 2-deoxy-d-glucose, metabolic inhibitors that function by depleting cells of energy. Oligomycin blocks oxidative phosphorylation, making cells more dependent on glycolysis and consequently more sensitive to inhibitors such as 2-deoxy-d-glucose.^90,91^ We observed that following cell energy depletion, on incubation with Ru-PDC_3_, emission was confined to the cell membrane (Figure S42) and confocal *z*-scanning throughout the cells confirmed there was no emission from the cell interior. Thus, we conclude that uptake occurs via an energy-dependent process, most likely endocytosis. To evaluate, then if the punctate distribution of the probe is due to endosomal entrapment, we evaluated the colocalization of the complex with Rab7a-GFP, a commercial dye used to stain late endosomes.^92^

Representative colocalization studies are shown in Figure 6, where Ru-PDC_3_ (shown in red) was found to colocalize to an extent with Rab7a-GFP (in yellow), evident by the overlap regions (in orange) and Pearson’s coefficient value of 0.73, though it is clear that Ru-PDC_3_ is not solely confined to the endosomes. Late endosomes serve as a sorting station for internalized extracellular molecules and can fuse with lysosomes as part of the endocytic pathway.^93^ We thus evaluated the colocalization of emission of Ru-PDC_3_ with LysoTracker Green, a lysosomal staining dye, and representative colocalization images are shown in Figure S43; Pearson’s coefficient value of 0.51 ± 0.03 was obtained, suggesting partial localization of the complex in lysosomes under these conditions. The membrane bilayer of endosomes and lysosomes creates an enclosed protected environment, which allows the luminescent-based tracking of Ru-PDC_3_ in cells. In addition to their conventional roles, both endosomes and lysosomes can release their contents to the cytoplasm or to the extracellular space. The distribution of the complex remained similar following incubation for an additional 24 h in the absence of light. Co-staining with LysoTracker Green (Figure S44) under these conditions indicated a slightly higher degree of lysosomal colocalization (*r* = 0.56 ± 0.01). Further co-staining studies were carried out with a mitochondrial staining dye (BioTracker Mitochondria 488 dye), which confirmed that the Ru-PDC_3_ complex does not localize to the mitochondria (Figure S45). Colocalization studies and r coefficients suggest that the staining observed originates primarily from the dye residing in endosomal vesicles and lysosomal organelles but potentially also in cytoplasmic compartments below the resolution of a conventional confocal microscope (Figures 6, S43, and S44).

Cytotoxicity studies using the alamarBlue assay showed that the Ru-PDC3 complex was mildly toxic toward the A549 cell line with an IC_50_ of 85 ± 10 μM (Figure S46). Although extensive distribution of the complex within cells was observed, the low cytotoxicity is tentatively attributed to the significant endosomal entrapment of the complex under these conditions. By comparison, a series of ligand (PDC)-based derivatives in A549 cells showed moderate toxicity over a 72 h incubation with IC_50_ values ranging from 16 μM to above 100 μM.^94^^94^

## Emission Lifetime Imaging

**Figure 7:**
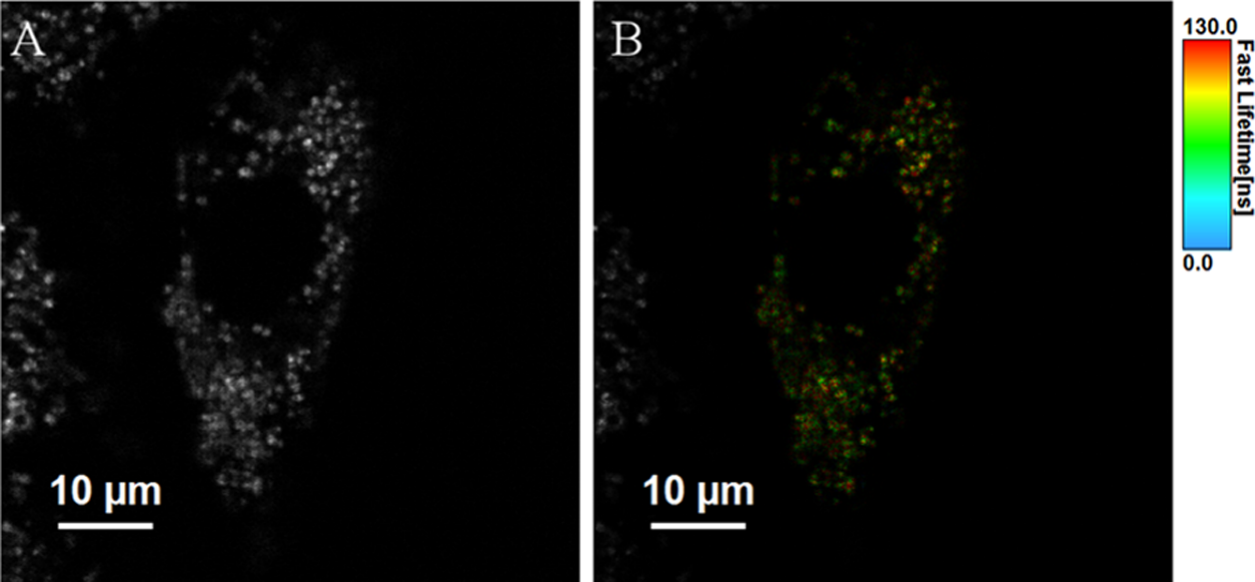
Representative (A) luminescence intensity (gray scale) and (B) lifetime color map images of live A549 cells treated with Ru-PDC3 at 50 μM for 24 h. The PLIM images were acquired using the 405 nm excitation laser line.

Phosphorescence lifetime imaging microscopy (PLIM) imaging was used to examine the lifetime distribution of Ru-PDC_3_ in A549 cells post 24 h of incubation. In all cases, the emission decays from the acquired PLIM images were best fit to a triexponential model where the short lifetime component is attributed to background scatter and/or reflectance. Figure 7 shows the intensity image and the corresponding false-color PLIM image of Ru-PDC_3_ in live A549 cells. As expected, the punctate distribution of the emission from the complex matches the confocal fluorescence imaging. As shown in PLIM images (Figures 7B and S47), the lifetime distribution is not uniform and shows significant variation between the spherical bodies that are labeled and lifetimes within a single A549 cell. For the PLIM image (Figure 7B), the complex exhibited an average lifetime of τ_1_ 120.4 ± 3.7 ns and τ_2_ 22.7 ± 1.2 ns. Interestingly, variation in the lifetime distribution was also observed in PLIM images acquired for different A549 cells. For example, Figure S49 shows the PLIM image for Ru-PDC_3_ in a different single live A549 cell. Here, the complex exhibited average lifetimes of 50.5 ± 1.8 and 9.51 ± 0.57 ns. The intercell variation is unexpected but may indicate that the lifetime is influenced by the life phase of the cell. These findings show that Ru-PDC3 can provide lifetime discrimination in imaging in cells and therefore could be used as a highly responsive probe for cell PLIM mapping.

Overall, the relatively short lifetimes observed from PLIM indicate that the complex is in a relatively aqueous-exposed environment within the cell. The lifetimes correspond fairly closely with the values of τ_2_ and τ_3_ from the parent when exposed to nucleic acid and given nuclear exclusion we attribute this to association with the endosomal membrane. There is no evidence of the long-lived lifetime component expected in G4 binding. In addition, based on the localization studies above, we conclude that the complex is likely emitting from the membrane of endosomes and lysosomes. The long- and short-lived components may reflect different modalities of membrane association, although the lifetime distribution appears to be uniform at each organelle and so may reflect the impact of the endosomal and lysosomal microenvironments on the emission of the complex. The latter is expected to be acidic and also contains a milieu of metals and redox-active species, which may result in the quenching of the excited state. Thus, overall, although unable to reach nucleic acid materials within the cell without further modification, our data shows that emission lifetimes from other components within the cell, presumably here membranous and proteinaceous structures, yield emission decays from FLIM that would be readily distinguishable from nucleic acid and ultimately from G4 structures. Our future focus is on modifications to the complex to promote targeting to the nucleus of the cell.

## Conclusions

The light switch capability of the well-known ruthenium tetrapyrido[3,2-*a*:2′,3′-*c*:3″,2″-*h*:2‴,3‴-*j*]phenazine (tpphz) complex was combined with a G4 selective ligand (Phen-DC3) to create a high-affinity reporter for G-quadruplex DNA. The complex shows complex absorption and emission behavior. Computation shows overlapping contributions from three charge-transfer transitions originating from the Ru(II) center to the phen ligand, the terminal methylquinolinium, and to tetrapyridophenazine of the PDC3 ligand. This is supported by resonance Raman data, which shows that under a 473 nm excitation, vibrational modes from all of the ligand systems are enhanced. The complex shows the light switch effect; emission is extinguished in water and switched on in acetonitrile or in association with the nucleic acid. From emission titrations, the complex shows particularly high affinity for G4 quadruplex DNA, although the affinity was G4 identity-dependent with *K*_a_ over an order of magnitude greater for K-ras and 22AG compared with ctDNA. The emission intensity and average emission lifetime are also greatly enhanced by binding to these two G4 structures. Interestingly, the resonance Raman spectroscopy shows a small but important change in quadruplex binding; the vibrational mode at 1534 cm^–1^ isolated on the phenazine is lost on G4 binding but not on association with other nucleic acids.

Critically, and unexpectedly, the complex was found to be a potent G4 disrupter. Circular dichroism revealed that across all G4s studied, a dramatic loss of G4 ellipticity occurred, which was particularly prevalent for K-ras and TBA, both of which showed a significantly induced CD at the metal complex absorbance. Furthermore, interestingly, the disruption does not correlate with the binding affinity or enhancement in emission intensity.

The complex, as a chloride salt, is water-soluble and was found to be readily cell-permeable through an activated uptake mechanism. The complex was found in A549 cells to be localized to a significant degree, although not exclusively, to endosomes and lysosomes in live cells, consistent with an endocytotic uptake mechanism. It showed strong emission from within the cells and based on emission lifetime imaging the emission switch is observed due to association with the organelle membrane rather than nucleic acid materials. Current studies focus on strategies to drive this interesting G4 disrupter to the nucleus.

## Experimental Section

### Materials and Methods

Absorbance measurements were performed using a Jasco V670 spectrophotometer; data was manipulated using Jasco Spectra Manager software and MS Excel. Emission titration spectra were obtained using a Varian Cary Eclipse Fluorimeter. Slit widths were set to 20 nm for all DNAs with the exception of emission studies using the INTER quadruplex where 10 nm was used due to greater emissivity. Lifetime measurements were performed on a PicoQuant FluoTime 100 Compact FLS TCSPC system using a 450 nm pulsed laser from a PicoQuant PDL800-B source and an external Thurlby Thandar Instruments TGP110 10 MHz pulse generator to enable the acquisition of long lifetime data. Data was collected from up to 10,000 counts, and decay curves were analyzed using PicoQuant Fluofit software and tail-fit statistical modeling. Deaeration was performed by bubbling samples with argon for 15 min before measurement. Raman spectroscopy was carried out on a Horiba LabRam instrument, using diode laser excitation at 785 or 473 nm. Circular dichromism measurements were performed on a Chirascan Series, and data manipulation was performed using the Global 3 Analysis Software and MS Excel. Comparative emission measurements of Ru-PDC3 bound to G4s were recorded on a Clariostar Plus plate reader in triplicate. High-resolution mass spectrometry (HR-MS) was performed by the HR-MS facility, Trinity College Dublin. Analytical HPLC was performed on a Varian 940-LC Liquid Chromatograph using an Agilent Zorbax Pursuit XRs C18 column (5 μm, 4.6 × 250 mm^2^). Samples were prepared in distilled water; gradient elution was performed with a flow rate of 1 mL/min starting with a 0.1% trifluoroacetic acid (TFA) in the H_2_O/MeCN mobile phase starting with 95/5 increasing linearly to 50/50 over 20 min. Peak detection was performed using a PDAD at 440 nm.

Stock concentrations of Ru-PDC3 were stored as PF_6_^–^ salts in MeCN or as Cl^–^ salts in deionized water at a concentration of 5 mM.

Oligonucleotides were purchased from Eurofins Genomics and stored at −20 °C. The preparation of G4s was performed similarly to previous reports.^86^ A KPi buffer (10 mM potassium phosphate, 100 mM KCl) was used to anneal the quadruplex for all measurements. Quadruplexes were annealed at 95 °C for 5 min before cooling slowly to room temperature. Fluorescent and absorbance titration experiments were performed in tandem using a stock concentration of a 1 mM quadruplex strand concentration.

Chemicals were purchased from either Sigma-Aldrich (Merck) or Fluorochem and used without further purification. ^1^H NMR spectra were recorded on a 600 MHz Bruker spectrometer. ^1^H NMR spectra were processed using the Bruker Topspin software. The complexes [Ru(phen)_2_(NitroAmino-phen)]^2+^ and [Ru(phen)_2_ (Diamino-phen)]^2^ were synthesized as described by Gillard et al.^95^

#### Raman Studies

Resonance Raman spectroscopy was carried out on Ru-PDC3 (100 μM) in KPi. Instrument calibration was performed prior to measurement. Ru-PDC3 was measured in the presence of DNA with a 473 nm laser with 20 accumulations per measurement. Measurements were repeated at least three times. Spectra in this section are representative spectra. Baseline correction and peak picking were completed using NGS LabSpec5 software.

#### DNA Titration Studies

DNA binding studies were performed using a procedure adapted from a literature protocol.^48^ Aliquots of DNA from a 1 mM stock solution were titrated into a 10 μM solution of Ru-PDC3 in KPi buffer. After addition, the solution was mixed with and incubated before luminescence measurements. Each titration was repeated at least three times. Addition of DNA was continued until saturation was evident. Measurements were performed with a 20 mm slit width with the exception of INTER G4 due to high emissivity, where 10 mm was used. Average values for *K*_b_ and *n* were obtained by fitting results to the model described by Carter et al. with modifications by Poulsen et al.^72,96^ The model is outlined in eqs 1 and 2. MS Solver was used to calculate the best fit while minimizing the sum of square residuals
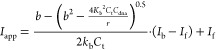
1
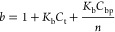
2where *I*_app_ is the apparent intensity of a given binding ratio, *r* = [DNA]/[Ru]; *I*_b_ is the intensity at saturation; *K*_b_ is the binding constant; *C*_t_ is the ruthenium concentration; *C*_dna_ is the total concentration of DNA by the G4 strand equivalent, base pair, or base for quadruplexes, dsDNA, and ssDNA, respectively; and *n* is the binding site size.

#### Circular Dichroism

Circular dichroism was performed using a G4 DNA solution annealed at 5 μM in KPi buffer. Titrations were repeated at least three times; each measurement presented is the average of two accumulations. Spectra were recorded over a window from 200 to 550 nm. Ru-PDC3 was titrated (2.5–50 μM) in each sample and measured after at least 5 min to allow binding to complete (following prior confirmation that this length of time was sufficient to see no additional changes).

#### Cell Culture

The Ru-PDC3 parent complex was studied in a lung carcinoma cancer cell line (A549). The cells were subcultured using Dulbecco’s modified Eagle’s medium supplemented with 10% fetal bovine serum (FBS) and 1% penicillin–streptomycin and were grown at 37 °C with 5% CO_2_.

#### Uptake Studies of Ru-PDC3

A549 cells were seeded at 8 × 10^4^ cells in 35 mm glass-bottom culture dishes (Ibidi, Germany) and were allowed to grow for 48 h at 37 °C with 5% CO_2_. Cells were treated with Ru-PDC3 at the desired concentration using a dye stock solution of 1 mM in phosphate-buffered saline (PBS). Following the desired incubation time, at 37 °C at 5% CO_2_, the cells were washed twice with supplemented PBS (1.1 mM MgCl_2_ and 0.9 mM CaCl_2_). The treated cells were imaged directly using a Leica TCS DMi8 confocal microscope (63× oil immersion objective lens) with a heated stage at 37 °C. A 475 nm white light laser was used to excite Ru-PDC3, and the emission range was set to between 550 and 800 nm. In the metabolic inhibitor study, the cells were pretreated with 5 μM of oligomycin and 50 mM of 2-deoxy-d-glucose for 40 min and the contact solution was replaced with Ru-PDC3 at 50 μM/24 h in fresh media.

Rab7a-GFP dye, used to stain late endosomes, was added to the cells and incubated overnight at 37 °C prior to the addition of the ruthenium complex. Rab7a-GFP was excited at 488 nm, and emission was collected between 490 and 540 nm. LysoTracker Green DND 26, used for staining lysosomes, was added at 60 nM and incubated for 30 min prior to imaging (λ_exc_ 504 nm, λ_em_ range: 510–515 nm). BioTracker 488 Green (100 nM; 25 min), used to stain mitochondria, was excited at 488 nm, and emission was collected between 510 and 520 nm. A nuclear staining dye was added (DRAQ7; 3 μM) during uptake studies to distinguish intact live cells from permeabilized/dead cells. The 633 nm laser was used to excite DRAQ7, and emission was collected between 650 and 750 nm.

#### Cytotoxicity Studies

The alamarBlue assay (Promocell GmbH) was used to assess the cell viability of A549 cells treated with the Ru-PDC3 complex. The cells were seeded in flat-bottomed culture-treated 96-well plates at 10^4^ cells in 100 μL per well for 24 h at 37 °C with 5% CO_2_. The complex was added at concentrations of 100, 75, 50, 25, 10, and 5 μM (*n* = 3) and incubated for 24 h prior to the addition of the Resazurin reagent (10% v/v) for 6 h at 37 °C in the absence of light. Absorbance readings were carried out at 570 and 600 nm (corrected for background subtraction) using a CLARIOstar (plus) (v 5.70) plate reader. The viability assay was performed in triplicate.

#### Phosphorescence Lifetime Imaging Microscopy (PLIM)

Live A549 cells were prepared and treated with Ru-PDC3. Luminescence lifetime imaging was carried out using a PicoQuant 100 system attached to a Leica TCS inverted (DMi8) confocal microscope using a 63× oil immersion objective. Each sample was acquired for 120 s with a 512 × 512 resolution using a 405 nm laser line. Data was analyzed using PicoQuant SymphoTime software.

## Supporting Information Available

Synthetic procedures and characterization (including ^1^H NMR and mass spectra); HPLC chromatograms; emission spectra with titrated DNA and binding curves; calculated binding constants; lifetime decays of Ru-PDC3 bound to DNA; circular dichroism of G4 DNA titrated with Ru-PDC3; Raman spectra of Ru-PDC3 bound to DNA; confocal and lifetime images; and cytotoxicity assays (PDF)

## References

[ref1] Bochman M. L.; Paeschke K.; Zakian V. A. DNA Secondary Structures: Stability and Function of G-Quadruplex Structures. Nat. Rev. Genet. 2012, 13, 770–780. 10.1038/nrg3296. 23032257 PMC3725559

[ref2] Spiegel J.; Adhikari S.; Balasubramanian S. The Structure and Function of DNA G-Quadruplexes. Trends Chem. 2020, 2, 123–136. 10.1016/j.trechm.2019.07.002. 32923997 PMC7472594

[ref3] Robinson J.; Raguseo F.; Nuccio S. P.; Liano D.; Di Antonio M. DNA G-Quadruplex Structures: More than Simple Roadblocks to Transcription?. Nucleic Acids Res. 2021, 49, 8419–8431. 10.1093/nar/gkab609. 34255847 PMC8421137

[ref4] Liu Y.; Zhu X.; Wang K.; Zhang B.; Qiu S. The Cellular Functions and Molecular Mechanisms of G-Quadruplex Unwinding Helicases in Humans. Front. Mol. Biosci. 2021, 8, 783889 10.3389/fmolb.2021.783889. 34912850 PMC8667583

[ref5] Di Antonio M.; Ponjavic A.; Radzevičius A.; Ranasinghe R. T.; Catalano M.; Zhang X.; Shen J.; Needham L.-M.; Lee S. F.; Klenerman D.; Balasubramanian S. Single-Molecule Visualization of DNA G-Quadruplex Formation in Live Cells. Nat. Chem. 2020, 12, 832–837. 10.1038/s41557-020-0506-4. 32690897 PMC7610488

[ref6] Summers P. A.; Lewis B. W.; Gonzalez-Garcia J.; Porreca R. M.; Lim A. H. M.; Cadinu P.; Martin-Pintado N.; Mann D. J.; Edel J. B.; Vannier J. B.; Kuimova M. K.; Vilar R. Visualising G-Quadruplex DNA Dynamics in Live Cells by Fluorescence Lifetime Imaging Microscopy. Nat. Commun. 2021, 12, 162 10.1038/s41467-020-20414-7. 33420085 PMC7794231

[ref7] Richter S. N.; Maggi S.; Mels S. C.; Palumbo M.; Freccero M. Binol Quinone Methides as Bisalkylating and DNA Cross-Linking Agents. J. Am. Chem. Soc. 2004, 126, 13973–13979. 10.1021/ja047655a. 15506758

[ref8] Di Antonio M.; Doria F.; Richter S. N.; Bertipaglia C.; Mella M.; Sissi C.; Palumbo M.; Freccero M. Quinone Methides Tethered to Naphthalene Diimides as Selective G-Quadruplex Alkylating Agents. J. Am. Chem. Soc. 2009, 131, 13132–13141. 10.1021/ja904876q. 19694465

[ref9] O’Hagan M. P.; Morales J. C.; Galan M. C. Binding and Beyond: What Else Can G-Quadruplex Ligands Do?. Eur. J. Org. Chem. 2019, 2019, 4995–5017. 10.1002/ejoc.201900692.

[ref10] Monchaud D. Quadruplex Detection in Human Cells. In Annual Reports in Medicinal Chemistry; Neidle S., Ed.; Quadruplex Nucleic Acids as Targets for Medicinal Chemistry; Academic Press, 2020; Chapter 5, Vol. 54, pp 133–160.

[ref11] Biffi G.; Tannahill D.; McCafferty J.; Balasubramanian S. Quantitative Visualization of DNA G-Quadruplex Structures in Human Cells. Nat. Chem. 2013, 5, 182–186. 10.1038/nchem.1548. 23422559 PMC3622242

[ref12] Hänsel-Hertsch R.; Beraldi D.; Lensing S. V.; Marsico G.; Zyner K.; Parry A.; Di Antonio M.; Pike J.; Kimura H.; Narita M.; Tannahill D.; Balasubramanian S. G-Quadruplex Structures Mark Human Regulatory Chromatin. Nat. Genet. 2016, 48, 1267–1272. 10.1038/ng.3662. 27618450

[ref13] Hänsel-Hertsch R.; Spiegel J.; Marsico G.; Tannahill D.; Balasubramanian S. Genome-Wide Mapping of Endogenous G-Quadruplex DNA Structures by Chromatin Immunoprecipitation and High-Throughput Sequencing. Nat. Protoc. 2018, 13, 551–564. 10.1038/nprot.2017.150. 29470465

[ref14] Hänsel-Hertsch R.; Simeone A.; Shea A.; Hui W. W. I.; Zyner K. G.; Marsico G.; Rueda O. M.; Bruna A.; Martin A.; Zhang X.; Adhikari S.; Tannahill D.; Caldas C.; Balasubramanian S. Landscape of G-Quadruplex DNA Structural Regions in Breast Cancer. Nat. Genet. 2020, 52, 878–883. 10.1038/s41588-020-0672-8. 32747825

[ref15] Kosiol N.; Juranek S.; Brossart P.; Heine A.; Paeschke K. G-Quadruplexes: A Promising Target for Cancer Therapy. Mol. Cancer 2021, 20, 40 10.1186/s12943-021-01328-4. 33632214 PMC7905668

[ref16] Paeschke K.; Bochman M. L.; Garcia P. D.; Cejka P.; Friedman K. L.; Kowalczykowski S. C.; Zakian V. A. Pif1 Family Helicases Suppress Genome Instability at G-Quadruplex Motifs. Nature 2013, 497, 458–462. 10.1038/nature12149. 23657261 PMC3680789

[ref17] Ribeyre C.; Lopes J.; Boulé J.-B.; Piazza A.; Guédin A.; Zakian V. A.; Mergny J.-L.; Nicolas A. The Yeast Pif1 Helicase Prevents Genomic Instability Caused by G-Quadruplex-Forming CEB1 Sequences In Vivo. PLoS Genet. 2009, 5, e1000475 10.1371/journal.pgen.1000475. 19424434 PMC2673046

[ref18] Makowski M. M.; Gräwe C.; Foster B. M.; Nguyen N. V.; Bartke T.; Vermeulen M. Global Profiling of Protein–DNA and Protein–Nucleosome Binding Affinities Using Quantitative Mass Spectrometry. Nat. Commun. 2018, 9, 1653 10.1038/s41467-018-04084-0. 29695722 PMC5916898

[ref19] Joachimi A.; Benz A.; Hartig J. S. A Comparison of DNA and RNA Quadruplex Structures and Stabilities. Bioorg. Med. Chem. 2009, 17, 6811–6815. 10.1016/j.bmc.2009.08.043. 19736017

[ref20] Manna S.; Srivatsan S. G. Fluorescence-Based Tools to Probe G-Quadruplexes in Cell-Free and Cellular Environments. RSC Adv. 2018, 8, 25673–25694. 10.1039/C8RA03708F. 30210793 PMC6130854

[ref21] Biffi G.; Di Antonio M.; Tannahill D.; Balasubramanian S. Visualization and Selective Chemical Targeting of RNA G-Quadruplex Structures in the Cytoplasm of Human Cells. Nat. Chem. 2014, 6, 75–80. 10.1038/nchem.1805. 24345950 PMC4081541

[ref22] Neidle S. Challenges in Developing Small-Molecule Quadruplex Therapeutics. In Quadruplex Nucleic Acids as Targets for Medicinal Chemistry; Neidle S., Ed.; Annual Reports in Medicinal Chemistry; Academic Press, 2020; Chapter 15, Vol. 54, pp 517–546.

[ref23] Awadasseid A.; Ma X.; Wu Y.; Zhang W. G-Quadruplex Stabilization via Small-Molecules as a Potential Anti-Cancer Strategy. Biomed. Pharmacother. 2021, 139, 111550 10.1016/j.biopha.2021.111550. 33831835

[ref24] Neidle S. Quadruplex Nucleic Acids as Novel Therapeutic Targets. J. Med. Chem. 2016, 59, 5987–6011. 10.1021/acs.jmedchem.5b01835. 26840940

[ref25] Salvati E.; Leonetti C.; Rizzo A.; Scarsella M.; Mottolese M.; Galati R.; Sperduti I.; Stevens M. F. G.; D’Incalci M.; Blasco M.; Chiorino G.; Bauwens S.; Horard B.; Gilson E.; Stoppacciaro A.; Zupi G.; Biroccio A. Telomere Damage Induced by the G-Quadruplex Ligand RHPS4 Has an Antitumor Effect. J. Clin. Invest. 2007, 117, 3236–3247. 10.1172/JCI32461. 17932567 PMC2000812

[ref26] Savva L.; Georgiades S. N. Recent Developments in Small-Molecule Ligands of Medicinal Relevance for Harnessing the Anticancer Potential of G-Quadruplexes. Molecules 2021, 26, 841 10.3390/molecules26040841. 33562720 PMC7914483

[ref27] Asamitsu S.; Obata S.; Yu Z.; Bando T.; Sugiyama H. Recent Progress of Targeted G-Quadruplex-Preferred Ligands Toward Cancer Therapy. Molecules 2019, 24, 429 10.3390/molecules24030429. 30682877 PMC6384606

[ref28] Tahara H.; Shin-ya K.; Seimiya H.; Yamada H.; Tsuruo T.; Ide T. G-Quadruplex Stabilization by Telomestatin Induces TRF2 Protein Dissociation from Telomeres and Anaphase Bridge Formation Accompanied by Loss of the 3′ Telomeric Overhang in Cancer Cells. Oncogene 2006, 25, 1955–1966. 10.1038/sj.onc.1209217. 16302000

[ref29] Burger A. M.; Dai F.; Schultes C. M.; Reszka A. P.; Moore M. J.; Double J. A.; Neidle S. The G-Quadruplex-Interactive Molecule BRACO-19 Inhibits Tumor Growth, Consistent with Telomere Targeting and Interference with Telomerase Function. Cancer Res. 2005, 65, 1489–1496. 10.1158/0008-5472.CAN-04-2910. 15735037

[ref30] Kench T.; Vilar R. Metal Complexes as G-Quadruplex Binders. In Quadruplex Nucleic Acids as Targets for Medicinal Chemistry; Neidle S., Ed.; Annual Reports in Medicinal Chemistry; Academic Press, 2020; Chapter 14, Vol. 54, pp 485–515.

[ref31] Wachter E.; Howerton B. S.; Hall E. C.; Parkin S.; Glazer E. C. A New Type of DNA “Light-Switch”: A Dual Photochemical Sensor and Metalating Agent for Duplex and G-Quadruplex DNA. Chem. Commun. 2014, 50, 311–313. 10.1039/C3CC47269H. 24226814

[ref32] Wachter E.; Moyá D.; Parkin S.; Glazer E. C. Ruthenium Complex “Light Switches” That Are Selective for Different G-Quadruplex Structures. Chem.—Eur. J. 2016, 22, 550–559. 10.1002/chem.201503203. 26560887 PMC4703525

[ref33] Verga D.; Hamon F.; Poyer F.; Bombard S.; Teulade-Fichou M.-P. Photo-Cross-Linking Probes for Trapping G-Quadruplex DNA. Angew. Chem., Int. Ed. 2014, 53, 994–998. 10.1002/anie.201307413. 24338872

[ref34] Lejault P.; Mitteaux J.; Sperti F. R.; Monchaud D. How to Untie G-Quadruplex Knots and Why?. Cell Chem. Biol. 2021, 28, 436–455. 10.1016/j.chembiol.2021.01.015. 33596431

[ref35] Kaluzhny D.; Ilyinsky N.; Shchekotikhin A.; Sinkevich Y.; Tsvetkov P. O.; Tsvetkov V.; Veselovsky A.; Livshits M.; Borisova O.; Shtil A.; Shchyolkina A. Disordering of Human Telomeric G-Quadruplex with Novel Antiproliferative Anthrathiophenedione. PLoS One 2011, 6, e27151 10.1371/journal.pone.0027151. 22102877 PMC3216923

[ref36] Weisman-Shomer P.; Cohen E.; Hershco I.; Khateb S.; Wolfovitz-Barchad O.; Hurley L. H.; Fry M. The Cationic Porphyrin TMPyP4 Destabilizes the Tetraplex Form of the Fragile X Syndrome Expanded Sequence d(CGG)n. Nucleic Acids Res. 2003, 31, 3963–3970. 10.1093/nar/gkg453. 12853612 PMC165968

[ref37] Waller Z. A. E.; Sewitz S. A.; Hsu S.-T. D.; Balasubramanian S. A Small Molecule That Disrupts G-Quadruplex DNA Structure and Enhances Gene Expression. J. Am. Chem. Soc. 2009, 131, 12628–12633. 10.1021/ja901892u. 19689109 PMC3037543

[ref38] Mitteaux J.; Lejault P.; Wojciechowski F.; Joubert A.; Boudon J.; Desbois N.; Gros C. P.; Hudson R. H. E.; Boulé J.-B.; Granzhan A.; Monchaud D. Identifying G-Quadruplex-DNA-Disrupting Small Molecules. J. Am. Chem. Soc. 2021, 143, 12567–12577. 10.1021/jacs.1c04426. 34346684

[ref39] Amjadi Oskouie A.; Abiri A. Refining Our Methodologies for Assessing Quadruplex DNA Ligands; Selectivity or an Illusion of Selectivity?. Anal. Biochem. 2021, 613, 113744 10.1016/j.ab.2020.113744. 32325085

[ref40] Shivalingam A.; Izquierdo M. A.; Marois A. L.; Vyšniauskas A.; Suhling K.; Kuimova M. K.; Vilar R. The Interactions between a Small Molecule and G-Quadruplexes Are Visualized by Fluorescence Lifetime Imaging Microscopy. Nat. Commun. 2015, 6, 8178 10.1038/ncomms9178. 26350962 PMC4579598

[ref41] Domarco O.; Kieler C.; Pirker C.; Dinhof C.; Englinger B.; Reisecker J. M.; Timelthaler G.; García M. D.; Peinador C.; Keppler B. K.; Berger W.; Terenzi A. Subcellular Duplex DNA and G-Quadruplex Interaction Profiling of a Hexagonal PtII Metallacycle. Angew. Chem., Int. Ed. 2019, 58, 8007–8012. 10.1002/anie.201900934. PMC6563712 31002438

[ref42] Gill M. R.; Garcia-Lara J.; Foster S. J.; Smythe C.; Battaglia G.; Thomas J. A. A Ruthenium(II) Polypyridyl Complex for Direct Imaging of DNA Structure in Living Cells. Nat. Chem. 2009, 1, 662–667. 10.1038/nchem.406. 21378959

[ref43] Berrones Reyes J.; Kuimova M. K.; Vilar R. Metal Complexes as Optical Probes for DNA Sensing and Imaging. Curr. Opin. Chem. Biol. 2021, 61, 179–190. 10.1016/j.cbpa.2021.02.007. 33784589

[ref44] Saadallah D.; Bellakhal M.; Amor S.; Lefebvre J.-F.; Chavarot-Kerlidou M.; Baussanne I.; Moucheron C.; Demeunynck M.; Monchaud D. Selective Luminescent Labeling of DNA and RNA Quadruplexes by π-Extended Ruthenium Light-Up Probes. Chem.—Eur. J. 2017, 23, 4967–4972. 10.1002/chem.201605948. 28124798

[ref45] Schindler J.; Traber P.; Zedler L.; Zhang Y.; Lefebvre J.-F.; Kupfer S.; Gräfe S.; Demeunynck M.; Chavarot-Kerlidou M.; Dietzek B. Photophysics of a Ruthenium Complex with a π-Extended Dipyridophenazine Ligand for DNA Quadruplex Labeling. J. Phys. Chem. A 2018, 122, 6558–6569. 10.1021/acs.jpca.8b05274. 30024161

[ref46] Mikek C. G.; Machha V. R.; White J. C.; Martin L. R.; West S. J.; Butrin A.; Shumaker C.; Gwin J. C.; Alatrash N.; MacDonnell F. M.; Lewis E. A. The Thermodynamic Effects of Ligand Structure on the Molecular Recognition of Mono- and Biruthenium Polypyridyl Complexes with G-Quadruplex DNA. Eur. J. Inorg. Chem. 2017, 2017, 3953–3960. 10.1002/ejic.201700789.

[ref47] Liao G.; Chen X.; Wu J.; Qian C.; Wang H.; Ji L.; Chao H. Novel Ruthenium(II) Polypyridyl Complexes as G-Quadruplex Stabilisers and Telomerase Inhibitors. Dalton Trans. 2014, 43, 7811–7819. 10.1039/C3DT53547A. 24699821

[ref48] Shi S.; Zhao J.; Geng X.; Yao T.; Huang H.; Liu T.; Zheng L.; Li Z.; Yang D.; Ji L. Molecular “Light Switch” for G-Quadruplexes and i-Motif of Human Telomeric DNA: [Ru(Phen)2(Dppz)]2+. Dalton Trans. 2010, 39, 2490–2493. 10.1039/B916094A. 20179840

[ref49] Shi S.; Huang H.-L.; Gao X.; Yao J.-L.; Lv C.-Y.; Zhao J.; Sun W.-L.; Yao T.-M.; Ji L.-N. A Comparative Study of the Interaction of Two Structurally Analogous Ruthenium Complexes with Human Telomeric G-Quadruplex DNA. J. Inorg. Biochem. 2013, 121, 19–27. 10.1016/j.jinorgbio.2012.12.011. 23333713

[ref50] Burke C. S.; Byrne A.; Keyes T. E. Targeting Photoinduced DNA Destruction by Ru(II) Tetraazaphenanthrene in Live Cells by Signal Peptide. J. Am. Chem. Soc. 2018, 140, 6945–6955. 10.1021/jacs.8b02711. 29767962

[ref51] Raza A.; Archer S. A.; Fairbanks S. D.; Smitten K. L.; Botchway S. W.; Thomas J. A.; MacNeil S.; Haycock J. W. A Dinuclear Ruthenium(II) Complex Excited by Near-Infrared Light through Two-Photon Absorption Induces Phototoxicity Deep within Hypoxic Regions of Melanoma Cancer Spheroids. J. Am. Chem. Soc. 2020, 142, 4639–4647. 10.1021/jacs.9b11313. 32065521 PMC7146853

[ref52] Jiang J.; Teunens T.; Tisaun J.; Denuit L.; Moucheron C. Ruthenium(II) Polypyridyl Complexes and Their Use as Probes and Photoreactive Agents for G-Quadruplexes Labelling. Molecules 2022, 27, 1541 10.3390/molecules27051541. 35268640 PMC8912042

[ref53] Weynand J.; Bonnet H.; Loiseau F.; Ravanat J.-L.; Dejeu J.; Defrancq E.; Elias B. Targeting G-Rich DNA Structures with Photoreactive Bis-Cyclometallated Iridium(III) Complexes. Chem.—Eur. J. 2019, 25, 12730–12739. 10.1002/chem.201902183. 31290208

[ref54] Weynand J.; Episkopou H.; Berre G. L.; Gillard M.; Dejeu J.; Decottignies A.; Defrancq E.; Elias B. Photo-Induced Telomeric DNA Damage in Human Cancer Cells. RSC Chem. Biol. 2022, 3, 1375–1379. 10.1039/D2CB00192F. 36544575 PMC9709782

[ref55] Archer S. A.; Raza A.; Dröge F.; Robertson C.; Auty A. J.; Chekulaev D.; Weinstein J. A.; Keane T.; Meijer A. J. H. M.; Haycock J. W.; MacNeil S.; Thomas J. A. A Dinuclear Ruthenium(Ii) Phototherapeutic That Targets Duplex and Quadruplex DNA. Chem. Sci. 2019, 10, 3502–3513. 10.1039/C8SC05084H. 30996941 PMC6430095

[ref56] Bevernaegie R.; Marcélis L.; Laramée-Milette B.; De Winter J.; Robeyns K.; Gerbaux P.; Hanan G. S.; Elias B. Trifluoromethyl-Substituted Iridium(III) Complexes: From Photophysics to Photooxidation of a Biological Target. Inorg. Chem. 2018, 57, 1356–1367. 10.1021/acs.inorgchem.7b02778. 29336560

[ref57] Holden L.; Burke C. S.; Cullinane D.; Keyes T. E. Strategies to Promote Permeation and Vectorization, and Reduce Cytotoxicity of Metal Complex Luminophores for Bioimaging and Intracellular Sensing. RSC Chem. Biol. 2021, 2, 1021–1049. 10.1039/D1CB00049G. 34458823 PMC8341117

[ref58] Martin A.; Byrne A.; Burke C. S.; Forster R. J.; Keyes T. E. Peptide-Bridged Dinuclear Ru(II) Complex for Mitochondrial Targeted Monitoring of Dynamic Changes to Oxygen Concentration and ROS Generation in Live Mammalian Cells. J. Am. Chem. Soc. 2014, 136, 15300–15309. 10.1021/ja508043q. 25265566

[ref59] Gkika K. S.; Noorani S.; Walsh N.; Keyes T. E. Os(II)-Bridged Polyarginine Conjugates: The Additive Effects of Peptides in Promoting or Preventing Permeation in Cells and Multicellular Tumor Spheroids. Inorg. Chem. 2021, 60, 8123–8134. 10.1021/acs.inorgchem.1c00769. 33978399 PMC8277133

[ref60] Rickling S.; Ghisdavu L.; Pierard F.; Gerbaux P.; Surin M.; Murat P.; Defrancq E.; Moucheron C.; Kirsch-De Mesmaeker A. A Rigid Dinuclear Ruthenium(II) Complex as an Efficient Photoactive Agent for Bridging Two Guanine Bases of a Duplex or Quadruplex Oligonucleotide. Chem.—Eur. J. 2010, 16, 3951–3961. 10.1002/chem.200902817. 20175157

[ref61] Poynton F. E.; Bright S. A.; Blasco S.; Clive Williams D.; Kelly J. M.; Gunnlaugsson T. The Development of Ruthenium(Ii) Polypyridyl Complexes and Conjugates for in Vitro Cellular and in Vivo Applications. Chem. Soc. Rev. 2017, 46, 7706–7756. 10.1039/C7CS00680B. 29177281

[ref62] Liao G.-L.; Chen X.; Ji L.-N.; Chao H. Visual Specific Luminescent Probing of Hybrid G-Quadruplex DNA by a Ruthenium Polypyridyl Complex. Chem. Commun. 2012, 48, 10781–10783. 10.1039/C2CC36039J. 23022988

[ref63] Heinemann F.; Karges J.; Gasser G. Critical Overview of the Use of Ru(II) Polypyridyl Complexes as Photosensitizers in One-Photon and Two-Photon Photodynamic Therapy. Acc. Chem. Res. 2017, 50, 2727–2736. 10.1021/acs.accounts.7b00180. 29058879

[ref64] Yang C.; Zhou Q.; Jiao Z.; Zhao H.; Huang C.-H.; Zhu B.-Z.; Su H. Ultrafast Excited State Dynamics and Light-Switching of [Ru(Phen)2(Dppz)]2+ in G-Quadruplex DNA. Commun. Chem. 2021, 4, 68 10.1038/s42004-021-00507-0. 36697709 PMC9814642

[ref65] Weynand J.; Diman A.; Abraham M.; Marcélis L.; Jamet H.; Decottignies A.; Dejeu J.; Defrancq E.; Elias B. Towards the Development of Photo-Reactive Ruthenium(II) Complexes Targeting Telomeric G-Quadruplex DNA. Chem.—Eur. J. 2018, 24, 19216–19227. 10.1002/chem.201804771. 30362627

[ref66] Chung W. J.; Heddi B.; Hamon F.; Teulade-Fichou M.-P.; Phan A. T. Solution Structure of a G-Quadruplex Bound to the Bisquinolinium Compound Phen-DC3. Angew. Chem., Int. Ed. 2014, 53, 999–1002. 10.1002/anie.201308063. 24356977

[ref67] De Cian A.; DeLemos E.; Mergny J.-L.; Teulade-Fichou M.-P.; Monchaud D. Highly Efficient G-Quadruplex Recognition by Bisquinolinium Compounds. J. Am. Chem. Soc. 2007, 129, 1856–1857. 10.1021/ja067352b. 17260991

[ref68] Liu Y.; Chouai A.; Degtyareva N. N.; Lutterman D. A.; Dunbar K. R.; Turro C. Chemical Control of the DNA Light Switch: Cycling the Switch ON and OFF. J. Am. Chem. Soc. 2005, 127, 10796–10797. 10.1021/ja052648n. 16076162

[ref69] Friedman A. E.; Chambron J. C.; Sauvage J. P.; Turro N. J.; Barton J. K. A Molecular Light Switch for DNA: Ru(Bpy)2(Dppz)2+. J. Am. Chem. Soc. 1990, 112, 4960–4962. 10.1021/ja00168a052.

[ref70] Sullivan B. P.; Salmon D. J.; Meyer T. J. Mixed Phosphine 2,2′-Bipyridine Complexes of Ruthenium. Inorg. Chem. 1978, 17, 3334–3341. 10.1021/ic50190a006.

[ref71] Park J. H.; Lee H. S.; Jang M. D.; Han S. W.; Kim S. K.; Lee Y.-A. Enantioselective Light Switch Effect of Δ- and Λ-[Ru(Phenanthroline)2 Dipyrido[3,2-a:2′, 3′-c]Phenazine]2+ Bound to G-Quadruplex DNA. J. Biomol. Struct. Dyn. 2018, 36, 1948–1957. 10.1080/07391102.2017.1345324. 28633570

[ref72] Poulsen B. C.; Estalayo-Adrián S.; Blasco S.; Bright S. A.; Kelly J. M.; Williams D. C.; Gunnlaugsson T. Luminescent Ruthenium Polypyridyl Complexes with Extended ‘Dppz’ like Ligands as DNA Targeting Binders and Cellular Agents. Dalton Trans. 2016, 45, 18208–18220. 10.1039/C6DT03792E. 27796397

[ref73] Ma D.-L.; Chan D. S.-H.; Fu W.-C.; He H.-Z.; Yang H.; Yan S.-C.; Leung C.-H. Discovery of a Natural Product-Like c-Myc G-Quadruplex DNA Groove-Binder by Molecular Docking. PLoS One 2012, 7, e43278 10.1371/journal.pone.0043278. 22912844 PMC3422278

[ref74] Ou A.; Schmidberger J. W.; Wilson K. A.; Evans C. W.; Hargreaves J. A.; Grigg M.; O’Mara M. L.; Iyer K. S.; Bond C. S.; Smith N. M. High Resolution Crystal Structure of a KRAS Promoter G-Quadruplex Reveals a Dimer with Extensive Poly-A π-Stacking Interactions for Small-Molecule Recognition. Nucleic Acids Res. 2020, 48, 5766–5776. 10.1093/nar/gkaa262. 32313953 PMC7261167

[ref75] Kumar R.; Chand K.; Bhowmik S.; Das R. N.; Bhattacharjee S.; Hedenström M.; Chorell E. Subtle Structural Alterations in G-Quadruplex DNA Regulate Site Specificity of Fluorescence Light-up Probes. Nucleic Acids Res. 2020, 48, 1108–1119. 10.1093/nar/gkz1205. 31912160 PMC7026600

[ref76] Shi S.; Geng X.; Zhao J.; Yao T.; Wang C.; Yang D.; Zheng L.; Ji L. Interaction of [Ru(Bpy)2(Dppz)]2+ with Human Telomeric DNA: Preferential Binding to G-Quadruplexes over i-Motif. Biochimie 2010, 92, 370–377. 10.1016/j.biochi.2010.01.003. 20096325

[ref77] Devereux S. J.; Poynton F. E.; Baptista F. R.; Gunnlaugsson T.; Cardin C. J.; Sazanovich I. V.; Towrie M.; Kelly J. M.; Quinn S. J. Caught in the Loop: Binding of the [Ru(Phen)2(Dppz)]2+ Light-Switch Compound to Quadruplex DNA in Solution Informed by Time-Resolved Infrared Spectroscopy. Chem.—Eur. J. 2020, 26, 17103–17109. 10.1002/chem.202002165. 32725823

[ref78] Rubio-Magnieto J.; Kajouj S.; Di Meo F.; Fossépré M.; Trouillas P.; Norman P.; Linares M.; Moucheron C.; Surin M. Binding Modes and Selectivity of Ruthenium Complexes to Human Telomeric DNA G-Quadruplexes. Chem.—Eur. J. 2018, 24, 15577–15588. 10.1002/chem.201802147. 30346057

[ref79] McQuaid K.; Abell H.; Gurung S. P.; Allan D. R.; Winter G.; Sorensen T.; Cardin D. J.; Brazier J. A.; Cardin C. J.; Hall J. P. Structural Studies Reveal Enantiospecific Recognition of a DNA G-Quadruplex by a Ruthenium Polypyridyl Complex. Angew. Chem., Int. Ed. 2019, 58, 9881–9885. 10.1002/anie.201814502. 30958918

[ref80] Shivalingam A.; Vyšniauskas A.; Albrecht T.; White A. J. P.; Kuimova M. K.; Vilar R. Trianguleniums as Optical Probes for G-Quadruplexes: A Photophysical, Electrochemical, and Computational Study. Chem.—Eur. J. 2016, 22, 4129–4139. 10.1002/chem.201504099. 26880483 PMC4991273

[ref81] Marchand A.; Gabelica V. Folding and Misfolding Pathways of G-Quadruplex DNA. Nucleic Acids Res. 2016, 44, 10999–11012. 10.1093/nar/gkw970. 27924036 PMC5159560

[ref82] D’Aria F.; D’Amore V. M.; Di Leva F. S.; Amato J.; Caterino M.; Russomanno P.; Salerno S.; Barresi E.; De Leo M.; Marini A. M.; Taliani S.; Da Settimo F.; Salgado G. F.; Pompili L.; Zizza P.; Shirasawa S.; Novellino E.; Biroccio A.; Marinelli L.; Giancola C. Targeting the KRAS Oncogene: Synthesis, Physicochemical and Biological Evaluation of Novel G-Quadruplex DNA Binders. Eur. J. Pharm. Sci. 2020, 149, 105337 10.1016/j.ejps.2020.105337. 32311457

[ref83] Reshetnikov R. V.; Sponer J.; Rassokhina O. I.; Kopylov A. M.; Tsvetkov P. O.; Makarov A. A.; Golovin A. V. Cation Binding to 15-TBA Quadruplex DNA Is a Multiple-Pathway Cation-Dependent Process. Nucleic Acids Res. 2011, 39, 9789–9802. 10.1093/nar/gkr639. 21893589 PMC3239185

[ref84] McQuaid K.; Hall J. P.; Baumgaertner L.; Cardin D. J.; Cardin C. J. Three Thymine/Adenine Binding Modes of the Ruthenium Complex Λ-[Ru(TAP) 2 (Dppz)] 2+ to the G-Quadruplex Forming Sequence d(TAGGGTT) Shown by X-Ray Crystallography. Chem. Commun. 2019, 55, 9116–9119. 10.1039/C9CC04316K. 31298665

[ref85] Renard I.; Grandmougin M.; Roux A.; Yang S. Y.; Lejault P.; Pirrotta M.; Wong J. M. Y.; Monchaud D. Small-Molecule Affinity Capture of DNA/RNA Quadruplexes and Their Identification in Vitro and in Vivo through the G4RP Protocol. Nucleic Acids Res. 2019, 47, 5502–5510. 10.1093/nar/gkz215. 30949698 PMC6582334

[ref86] Kejnovská I.; Renčiuk D.; Palacký J.; Vorlíčková M. CD Study of the G-Quadruplex Conformation. In G-Quadruplex Nucleic Acids; Yang D.; Lin C., Eds.; Methods in Molecular Biology; Humana: New York, 2019; Vol. 2035, pp 25–44. 10.1007/978-1-4939-9666-7_2 31444742

[ref87] Marchand A.; Granzhan A.; Iida K.; Tsushima Y.; Ma Y.; Nagasawa K.; Teulade-Fichou M.-P.; Gabelica V. Ligand-Induced Conformational Changes with Cation Ejection upon Binding to Human Telomeric DNA G-Quadruplexes. J. Am. Chem. Soc. 2015, 137, 750–756. 10.1021/ja5099403. 25525863

[ref88] Ghosh A.; Trajkovski M.; Teulade-Fichou M.-P.; Gabelica V.; Plavec J. Phen-DC3 Induces Refolding of Human Telomeric DNA into a Chair-Type Antiparallel G-Quadruplex through Ligand Intercalation. Angew. Chem. 2022, 134, e202207384 10.1002/ange.202207384. PMC9826182 35993443

[ref89] Coates C. G.; Jacquet L.; McGarvey J. J.; Bell S. E. J.; Al-Obaidi A. H. R.; Kelly J. M. Resonance Raman Probing of the Interaction between Dipyridophenazine Complexes of Ru(II) and DNA. J. Am. Chem. Soc. 1997, 119, 7130–7136. 10.1021/ja970064i.

[ref90] Pajak B.; Siwiak E.; Sołtyka M.; Priebe A.; Zieliński R.; Fokt I.; Ziemniak M.; Jaśkiewicz A.; Borowski R.; Domoradzki T.; Priebe W. 2-Deoxy-d-Glucose and Its Analogs: From Diagnostic to Therapeutic Agents. Int. J. Mol. Sci. 2020, 21, 234 10.3390/ijms21010234. PMC6982256 31905745

[ref91] Mookerjee S. A.; Nicholls D. G.; Brand M. D. Determining Maximum Glycolytic Capacity Using Extracellular Flux Measurements. PLoS One 2016, 11, e0152016 10.1371/journal.pone.0152016. 27031845 PMC4816457

[ref92] Gruenberg J.; Stenmark H. The Biogenesis of Multivesicular Endosomes. Nat. Rev. Mol. Cell Biol. 2004, 5, 317–323. 10.1038/nrm1360. 15071556

[ref93] Jeger J. L. Endosomes, Lysosomes, and the Role of Endosomal and Lysosomal Biogenesis in Cancer Development. Mol. Biol. Rep. 2020, 47, 9801–9810. 10.1007/s11033-020-05993-4. 33185829

[ref94] Masson T.; Landras Guetta C.; Laigre E.; Cucchiarini A.; Duchambon P.; Teulade-Fichou M.-P.; Verga D. BrdU Immuno-Tagged G-Quadruplex Ligands: A New Ligand-Guided Immunofluorescence Approach for Tracking G-Quadruplexes in Cells. Nucleic Acids Res. 2021, 49, 12644–12660. 10.1093/nar/gkab1166. 34875077 PMC8682774

[ref95] Gillard M.; Weynand J.; Bonnet H.; Loiseau F.; Decottignies A.; Dejeu J.; Defrancq E.; Elias B. Flexible RuII Schiff Base Complexes: G-Quadruplex DNA Binding and Photo-Induced Cancer Cell Death. Chem.—Eur. J. 2020, 26, 13849–13860. 10.1002/chem.202001409. 32484271

[ref96] Carter M. T.; Rodriguez M.; Bard A. J. Voltammetric Studies of the Interaction of Metal Chelates with DNA. 2. Tris-Chelated Complexes of Cobalt(III) and Iron(II) with 1,10-Phenanthroline and 2,2′-Bipyridine. J. Am. Chem. Soc. 1989, 111, 8901–8911. 10.1021/ja00206a020.

