# Supplementary material for: Selective, Disruptive Luminescent Ru(II) Polypyridyl Probes of G-Quadruplex
Source: Inorg Chem. 2023 Jan 27;62(5):2213–27. doi: 10.1021/acs.inorgchem.2c03903 (PMC9906756; doi:10.1021/acs.inorgchem.2c03903)
Supplement: Supplementary file 1 — ic2c03903_si_001.pdf [file ic2c03903_si_001.pdf]

Supporting Information for:

A Selective, Disruptive Luminescent Ru (II) Polypyridyl Probe of G-Quadruplex .

Lorcan Holden[a], Karmel S. Gkika[a], Christopher S. Burke[a.b], Conor Long[a], Tia E. Keyes[a*]

[a] National Centre for Sensor Research, School of Chemical Sciences, Dublin City University, Dublin 9, Co. Dublin, Ireland

[b] Current Address, School of Chemistry and Analytical and Biological Chemistry Research Facility, University College Cork, Co. Cork, Ireland

Corresponding author's email address: tia.keyes@dcu.ie

Table S1: Oligonucleotides used in this study

Name	Secondary structure	Sequence 5'->3'	Ref.
22AG	Mixed hybrid	AGGGTTAGGGTTAGGGTTAGGG	[1]
K-ras	Parallel	AGGGCGGTGTGGGATAGGGAA	[2]
TBA	Anti-parallel	GGTTGGTGTGGTTGG	[3]
Pu24T	Parallel	TGAGGGTGGTGAGGGTGGGGAAGG	[1]
INTER	Intermolecular	TAGGGTTA	[4]
ds17 A	Double Stranded	CCAGTTCGTAGTAACCC	[5]
ds17 B	Double Stranded	GGGTTACTACGAACTGG	[5]
ctDNA	Double stranded		-
ss17	Single Stranded	CCAGTTCGTAGTAACCC	[5]
A13	Single Stranded	AAAAAAAAAAAAA	-
T13	Single Stranded	TTTTTTTTTTTTT	-

Synthetic procedures

[Ru(phen)$_2$(Diamino-phen)]$^{2+}$

[Ru(phen)$_2$(Nirtroamino-phen)]$^{2+}$ (50 mg) and Pd/C (25 mg) were added to a 50:50 MeOH: EtOH solution and brought to reflux. Hydrazine hydrate (0.2 ml) was added, and the solution was refluxed for 1 hr before vacuum filtration. The filtrate was concentrated before purification on a silica gel column. Purified fractions were collected and reduced under pressure, redissolved in minimal MeCN before precipitation in a NH_4PF_6 solution to yield a red solid. (40 mg, 84% yield) ^{1}H NMR (600 MHz, CD_3CN): 8.60 (m, 4 H) 8.48 (dd, 2 H), 8.25 (t, 4 H), 8.03 (t, 2 H), 8.01 (t, 2 H), 7.78 (dd, 2 H), 7.60-7.66 (m, 4 H), 7.51 (dd, 2 H), 4.75 (s, 4 H).

1,10-phenanthroline-2,9-dicarboxylic acid-5,6-dione (1)

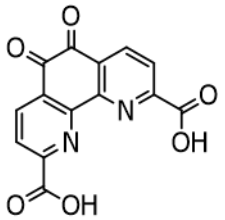

(1) Was synthesised as previously reported by Gislason et al.[11] Neocuproine (0.51 g) and KBr (0.42 g) were added to a RBF. Fuming sulfuric acid (5 ml) was added in portions to the round bottom flask followed by the dropwise addition of conc. nitric acid (2.5 ml). The solution was refluxed for 6 hours. After refluxing the solution was allowed to cool, poured over ice water (25 ml). The resulting yellow ppt. was filtered and washed with distilled water, diethyl ether. ^{1}H NMR (600 MHz) DMSO-d_6: 8.58 (d, J = 8.0 Hz 2H), 8.26 (d, J = 7.9 Hz, 2H).

5,6-dioxo-N2,N9-di(quinolin-3-yl)-5,6-dihydro-1,10-phenanthroline-2,9-dicarboxamide (2)

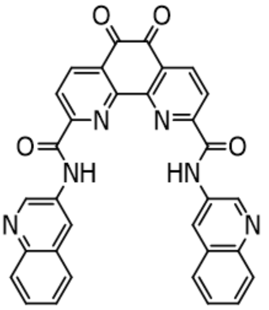

1,10-phenanthroline-2,9-dicarboxylic acid-5,6-dione (100 mg) was dissolved with anhydrous DMF in a N_2 purged round bottom flask. DIPEA (0.34 ml) was added followed by HATU (0.38 g) previously dissolved in DMF and allowed to stir for 30 minutes before addition of 3-aminoquinoline (0.97 g) previously dissolved in anhydrous DMF. The solution was stirred overnight. The orange ppt. was filtered and washed with minimal DMF, deionised water and diethyl ether. (82 mg, 44% yield) ^{1}H NMR (600 MHz): 11.69 (s, 2 H), 9.62 (d, J = 2.9 Hz, 2 H), 9.08 (d, J = 2.3 Hz, 2 H), 8.76 (d, J = 7.7 Hz, 2 H), 8.52 (d, J = 7.8 Hz, 2 H) 8.07-8.11 (m, 4 H), 7.76 (t, J = 8.0 Hz, 2 H), 7.67 (t, J = 8.0 Hz, 2 H). m/z calculated = 551.1473, Found 551.1464.

Phendione-DC3 (3)

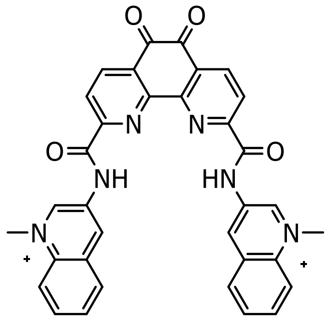

(2) (47mg) was dissolved in 30 ml of anhydrous dichloroethane and brought to reflux under N_2. Methyl trifluorosulfonate (0.3 ml) was added and the solution refluxed for 18 hrs. The yellow ppt. was filtered and washed with water and diethyl ether. (52 mg, 70% yield) ^{1}H NMR (600 MHz) 11.98 (s, 2 H), 10.20 (d, J = 2.3 Hz, 2 H), 9.80 (d, J = 1.6 Hz, 2 H), 8.83 (d, J = 8 Hz, 2 H) 8.61 (d, J = 8.1 Hz, 2 H), 8.54 (d, J = 8.8 Hz, 2H), 8.49 (d, J = 7.92 Hz, 2 H), 8.26 (t, J = 8.2 Hz, 2 H), 8.06 (t, J = 7.1 Hz, 2 H), 4.69 (s, 6 H) Meas. m/z calculated = 729.1385, Found 729.1384.

Ru-PDC3

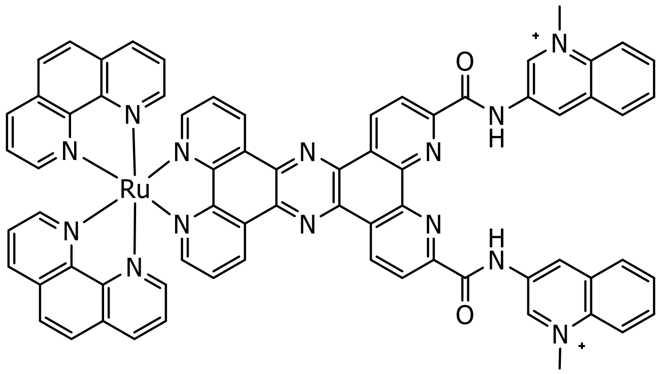

$[Ru(phen)_2(DAphen)](PF_6)_2$ (30 mg) and Phendione-DC3 (36 mg) were dissolved in dry MeCN. A catalytic amount of glacial acetic acid was added, and the solution was refluxed for 72 hrs under nitrogen. The solution was concentrated under vacuum and purified on silica column using a 80/20/1 ($MeCN/H_2O$/20% KNO_3) mobile phase. Product fractions were collected and combined. Sat. Ammonium hexafluorophosphate was added and the solution was concentrated under vacuum. The red ppt. was filtered and redissolved in minimal MeCN. The product was further precipitated in a sat. ammonium hexafluorophosphate solution. The orange product was filtered and washed with deionised water and diethyl ether. Yield = orange solid (41 mg, 73% yield). ^{1}H NMR (600 MHz, CD_3CN): 10.35 (d, J=7.8 Hz, 2 H), 10.14 (s, 2 H) 10.02 (d, J=7.8 Hz, 2 H) 9.51 (s, 2 H), 9.03 (d, J=8.0 Hz, 2 H), 8.67 (dd, J= 8.4, 1.1 Hz, 4 H), 8.43 (d, J= 9.0 Hz, 2 H), 8.20-8.34 (m, 12 H) 8.08 (d, J=5.0 Hz, 2 H) 8.04 (t, J= 7.8 Hz, 2 H), 7.90-7.95 (m, 2H), 7.67-7.74 (m, 4 H) 4.73 (s, 6 H). Meas. m/z $[M]^{3+}$ calculated = 405.0964, Found 405.0975.

Characterization Data

H^{1}NMR Spectra

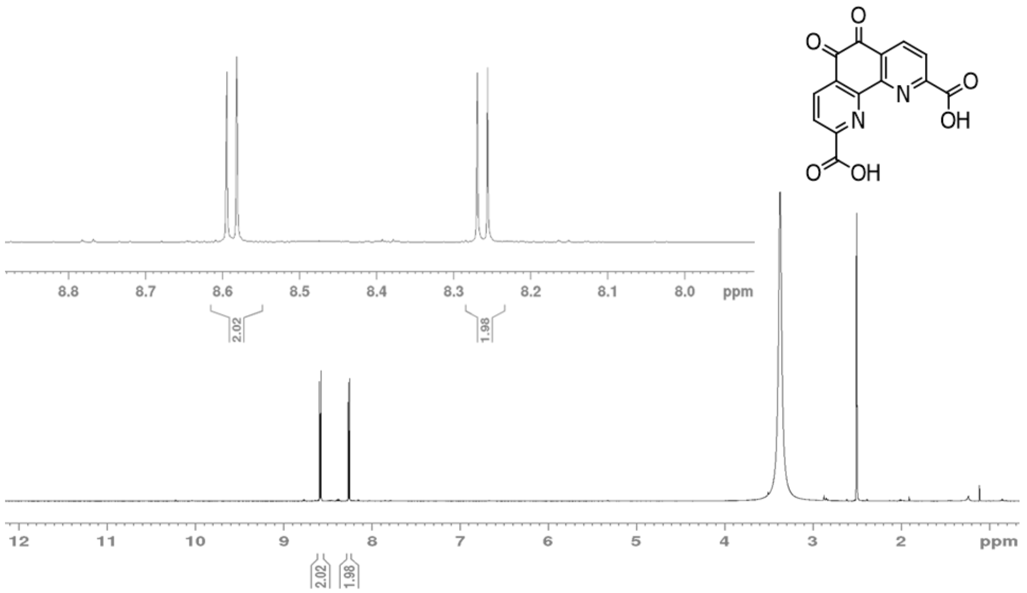

Figure S1: ^{1}H NMR (600 MHz) of 1,10-phenanthroline-2,9-dicarboxylic acid-5,6-dione in DMSO-d6. Inset shows the aromatic region.

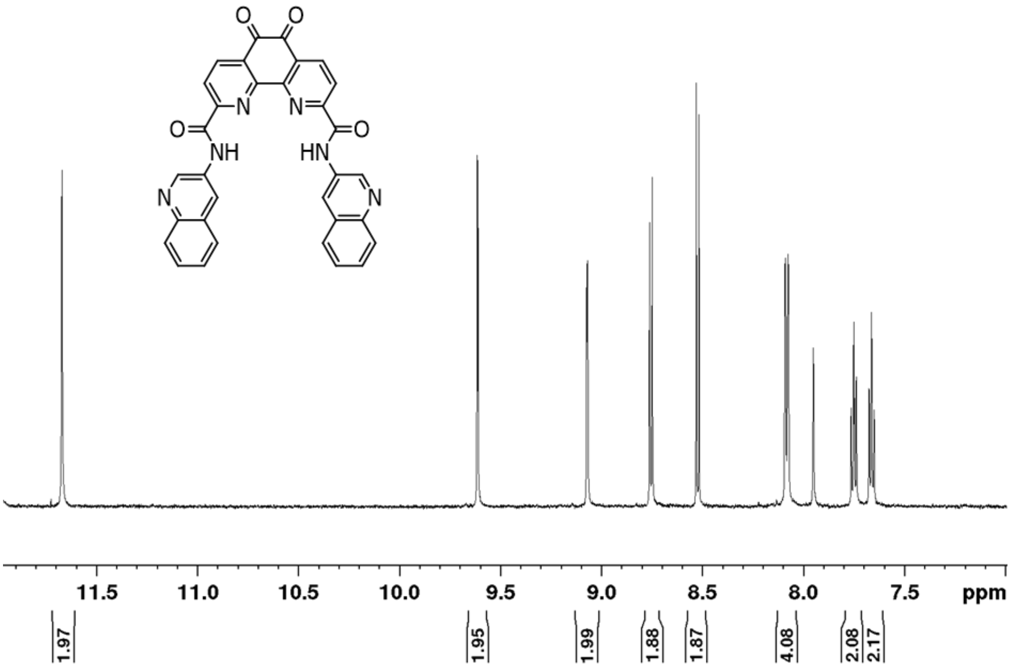

Figure S2: ^{1}H NMR (600 MHz) of 2 in DMSO-d6

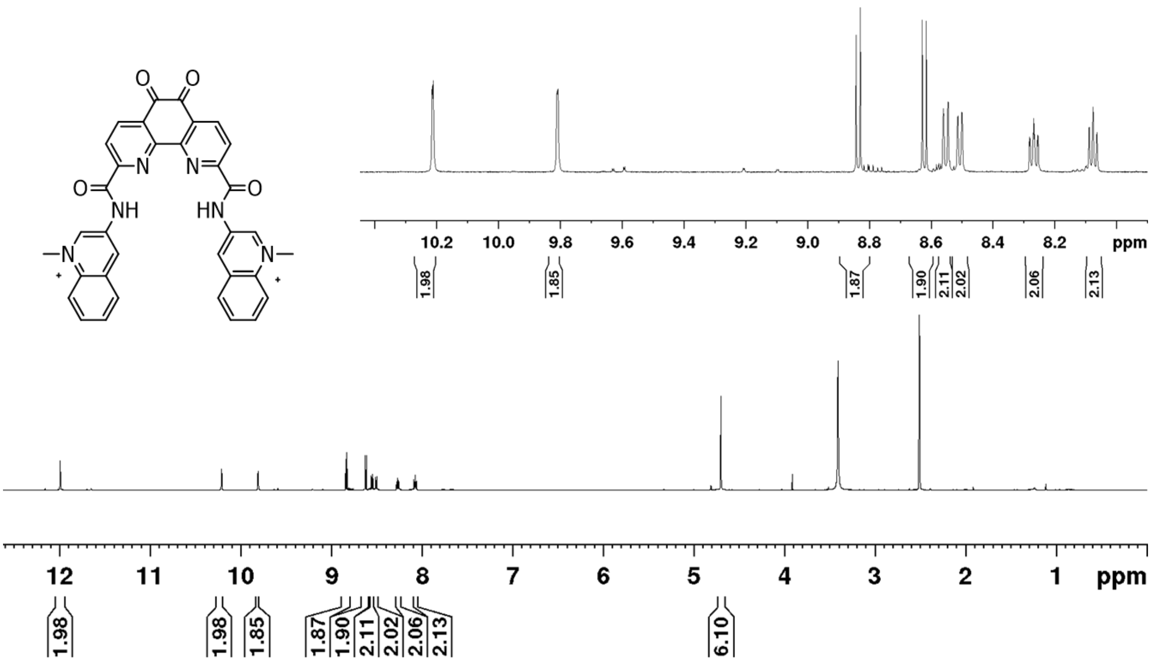

Figure S3: ^{1}H NMR (600 MHz) of 3 in DMSO-d6. Inset shows the aromatic region

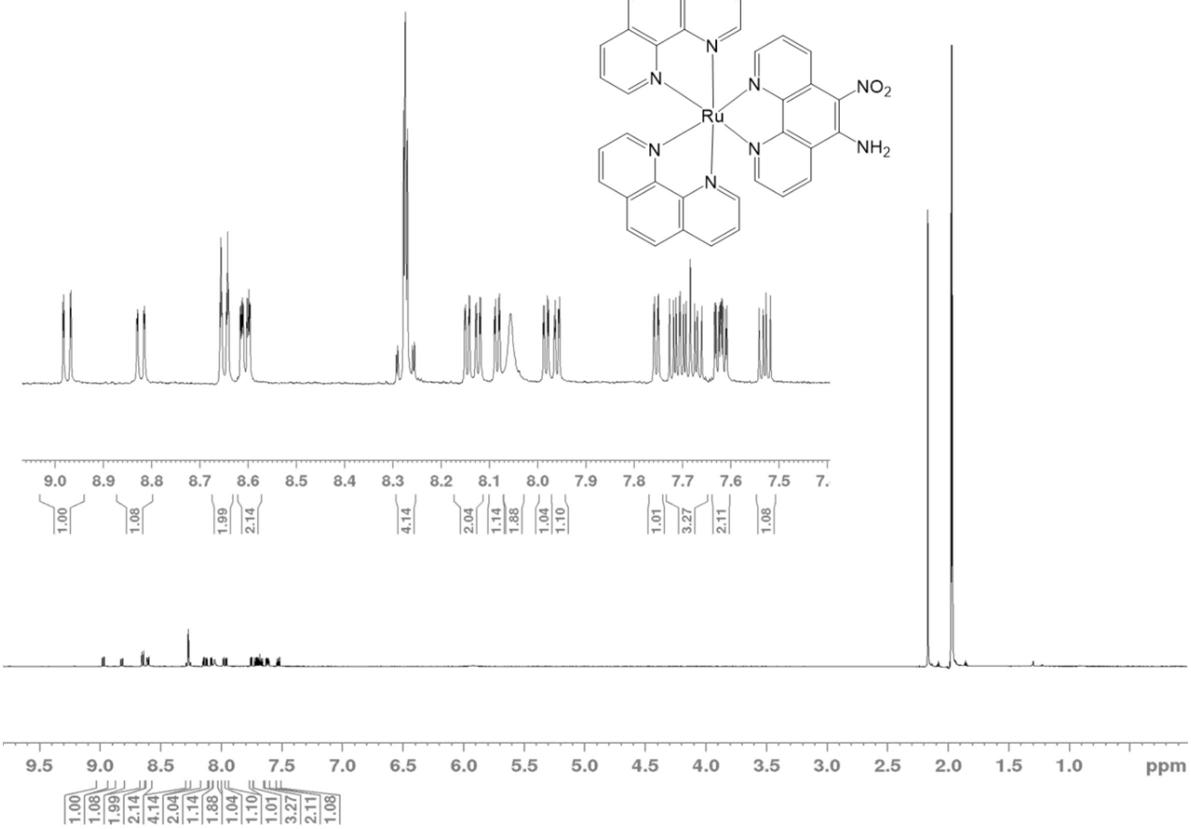

Figure S4: ^{1}H NMR (600 MHz) of [Ru(phen)(NitroAmino-phen)]^{2} in CD_3CN.

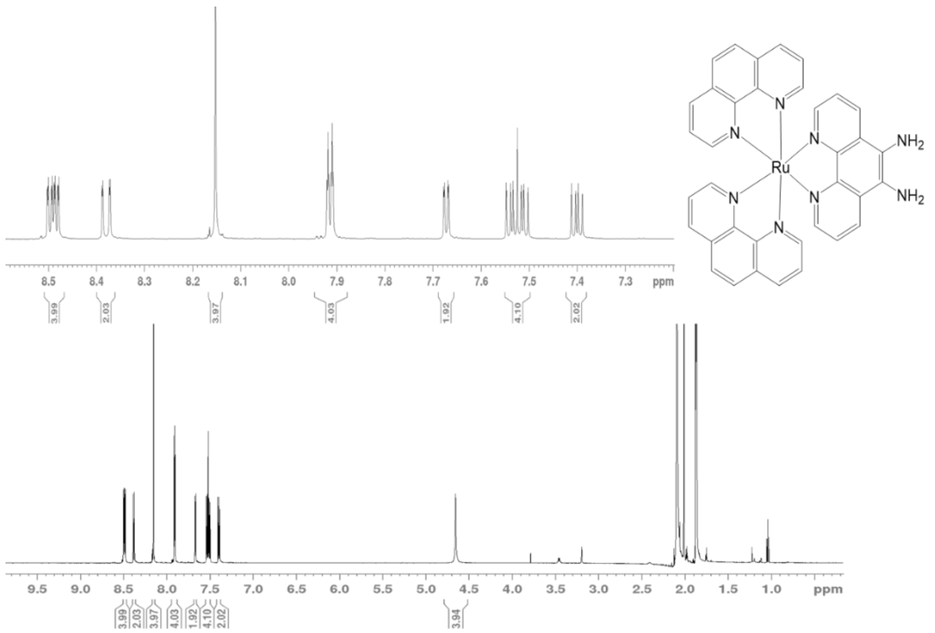

Figure S5: ^{1}H NMR (600 MHz) of [Ru(phen)(Diamino-phen)]$^{2=}$ in CD_3CN. Inset to show aromatic region.

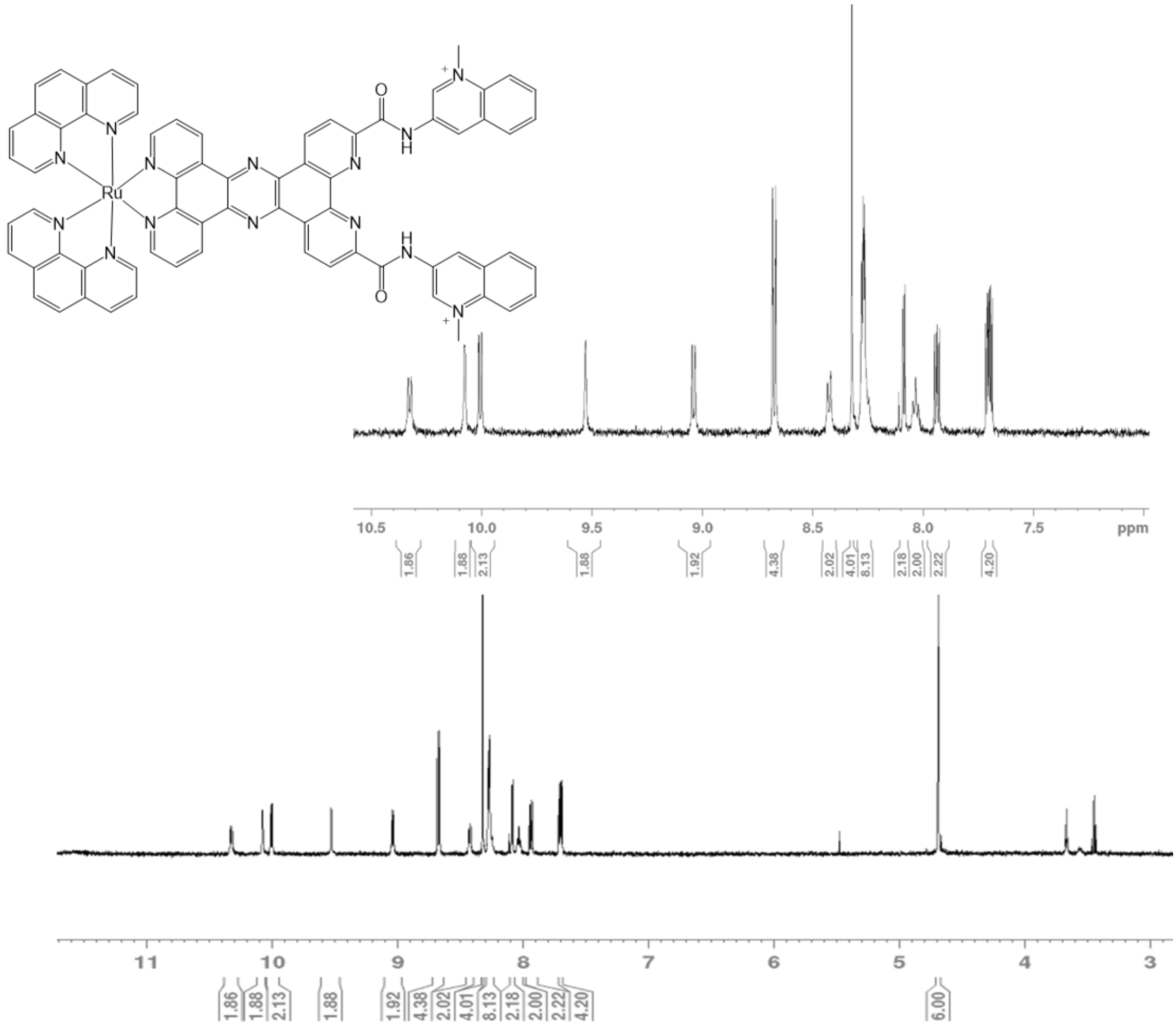

Figure S6: ^{1}H NMR (600 MHz) of Ru-PDC3 in CD_3CN. Inset to show aromatic region.

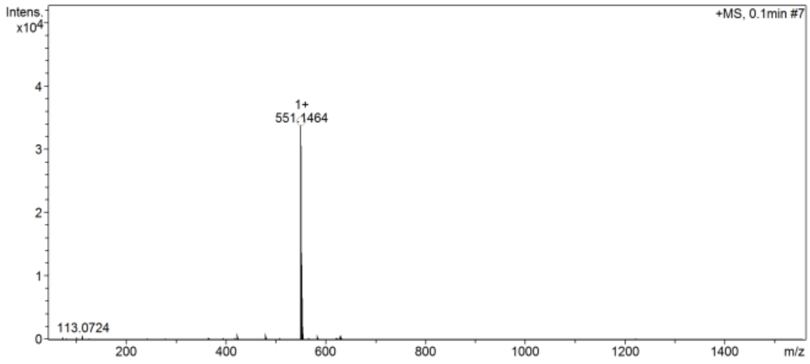

Meas. m/z	#	Ion Formula	m/z	err [mDa]	err [ppm]	rdb	N-Rule	e⁻ Conf	mSigma
551.146448	1	C32H19N6O4	551.146230	-0.2	-0.4	26.5	ok	even	4.7

Figure S7: Mass spectrum of (2).

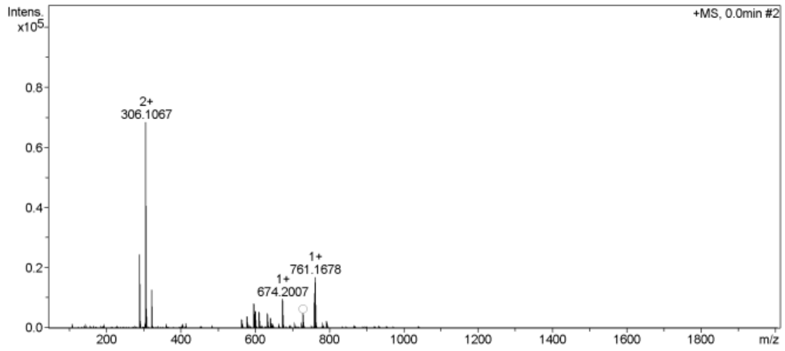

Meas. m/z	#	Ion Formula	m/z	err [mDa]	err [ppm]	rdb	N-Rule	e⁻ Conf	mSigma
729.138398	1	C35H24F3N6O7S	729.137379	1.0	1.4	25.5	ok	even	22.3

Figure S8: Mass spectrum of (3).

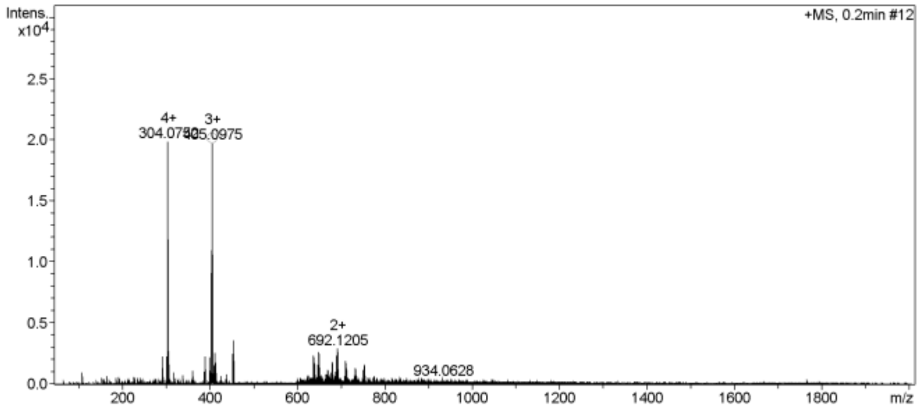

Meas. m/z	#	Ion Formula	m/z	err [mDa]	err [ppm]	rdb	N-Rule	e⁻ Conf	mSigma
405.097517	1	C70H45N14O2Ru	405.096523	1.0	2.5	56.0	-	odd	44.1

Figure S9: Mass spectrum of Ru-PDC3.

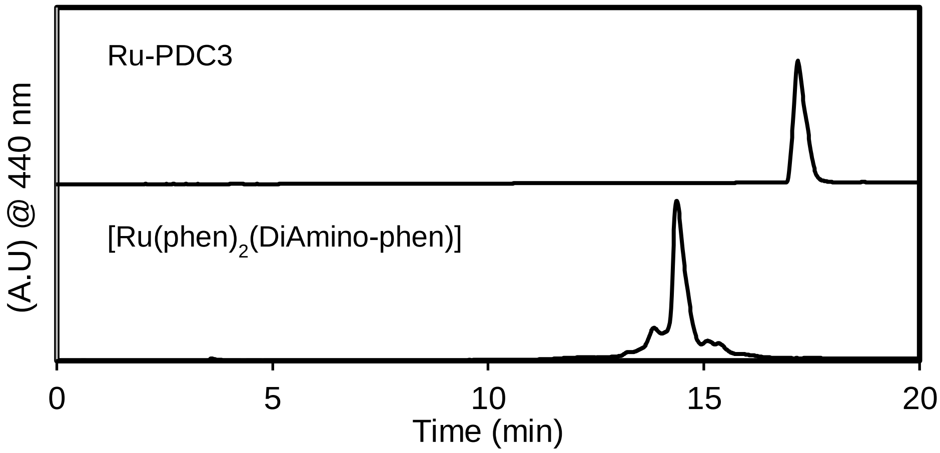

Figure S10: HPLC Chromatogram of Ru-PDC3 and [Ru(phen)$_2$(DiAmino-phen)]$^{2+}$ to indicate purity of final complex (RP-C18, 0.1% TFA in H_2O/MeCN).

Emission studies

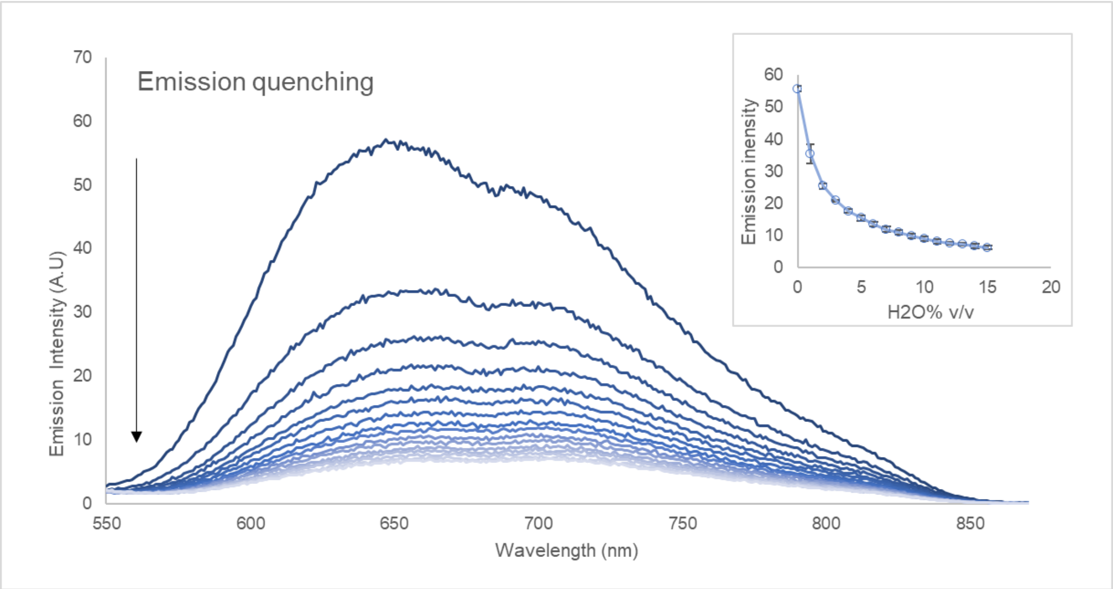

Figure S11: Change in emission intensity of 10 µM solution of Ru-PDC3 in MeCN upon addition of D.I Water

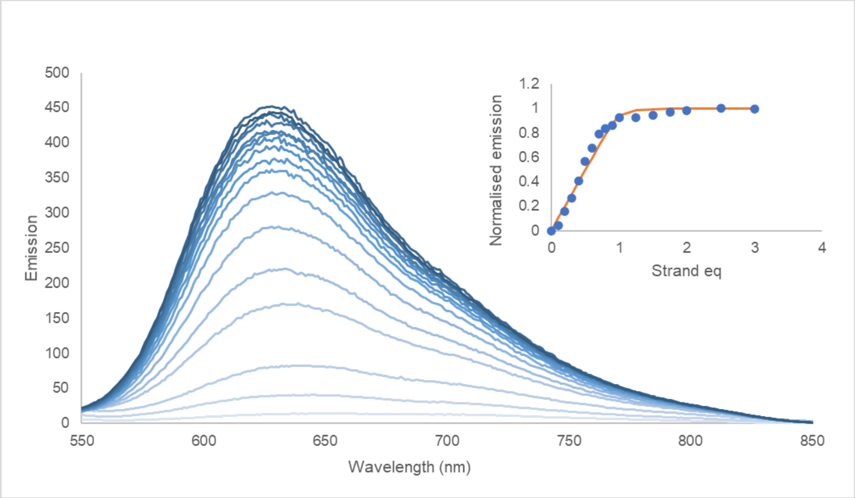

Figure S12: Change in emission of Ru-PDC3 (10 µM) with increasing concentration of 22AG (0-30 µM) and representative binding curve. Aqueous buffer solution is 10 mM potassium phosphate buffer with 100 mM KCl at pH7.

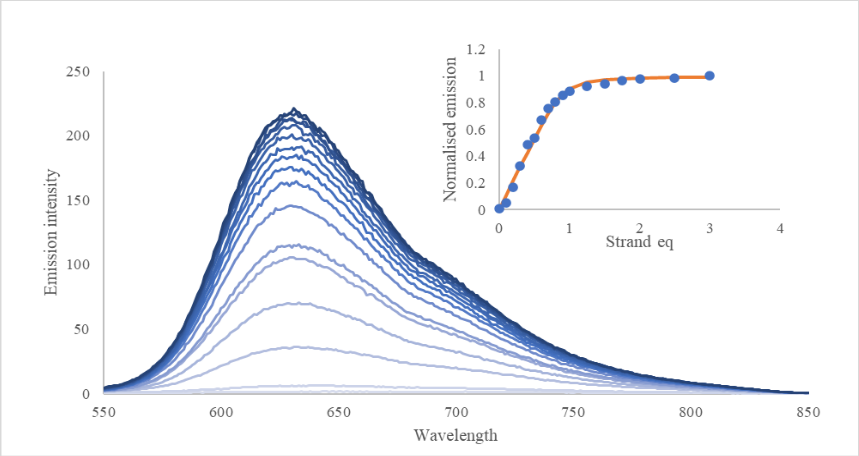

Figure S13: Change in emission of Ru-PDC3 (10 µM) with increasing concentration of INTER (0-30 µM) representative binding curve. Aqueous buffer solution is 10 mM potassium phosphate buffer with 100 mM KCl at pH7.

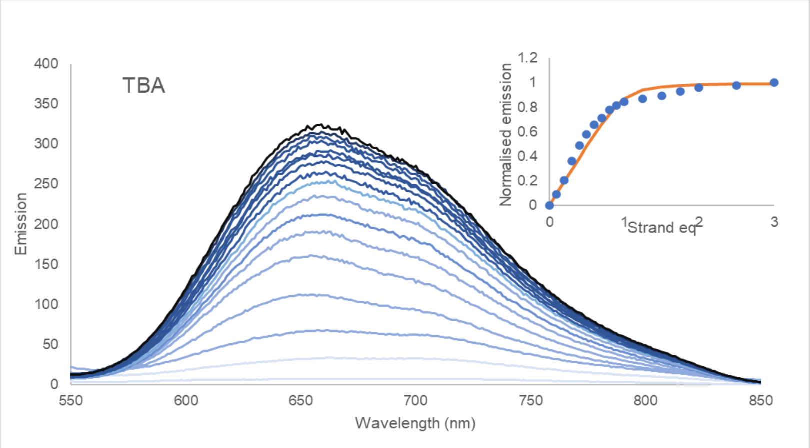

Figure S14: Change in emission of Ru-PDC3 (10 µM) with increasing concentration of TBA (0-30 µM) representative binding curve. Aqueous buffer solution is 10 mM potassium phosphate buffer with 100 mM KCl at pH7.

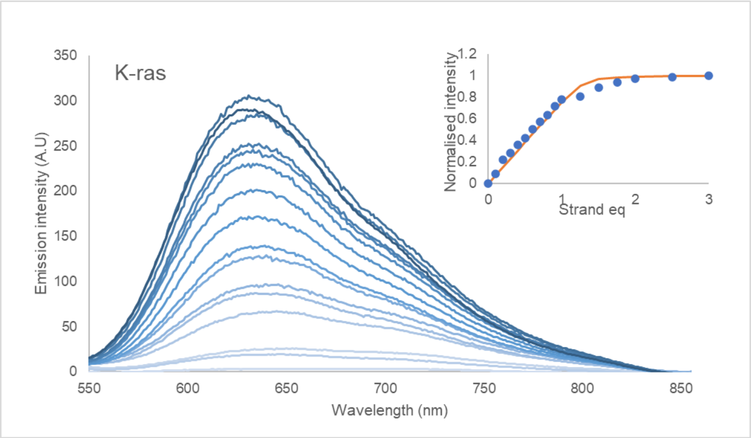

Figure S15: Change in emission of Ru-PDC3 (10 µM) with increasing concentration of KRAS(0-30 µM) representative binding curve. Aqueous buffer solution is 10 mM potassium phosphate buffer with 100 mM KCl at pH7.

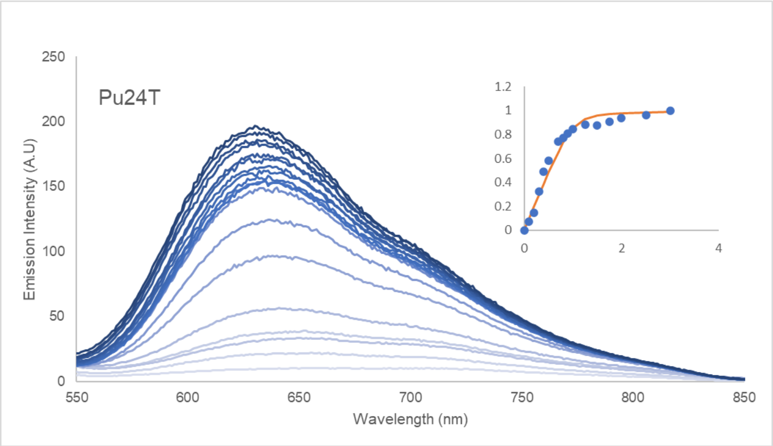

Figure S16: Change in emission of Ru-PDC3 (10 µM) with increasing concentration of Pu24T (0-30 µM) representative binding curve. Aqueous buffer solution is 10 mM potassium phosphate buffer with 100 mM KCl at pH7.

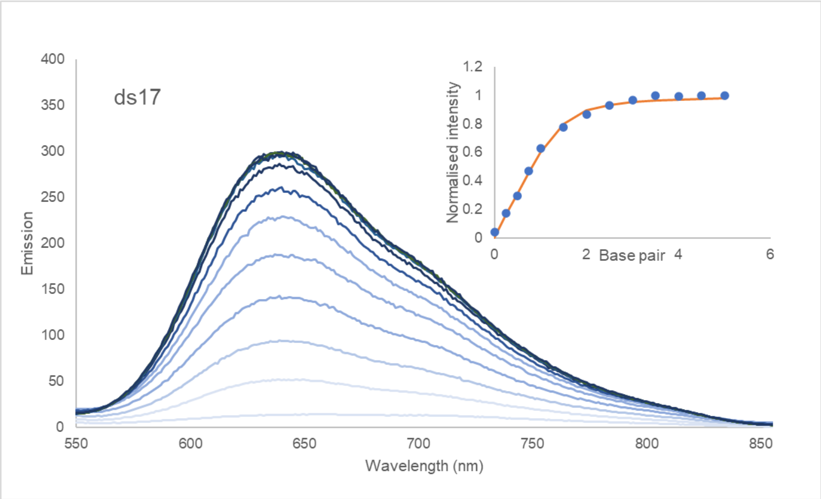

Figure S17: Change in emission of Ru-PDC3 (10 µM) with increasing concentration of ds17 (0-50 µM) representative binding curve. Aqueous buffer solution is 10 mM potassium phosphate buffer with 100 mM KCl at pH7.

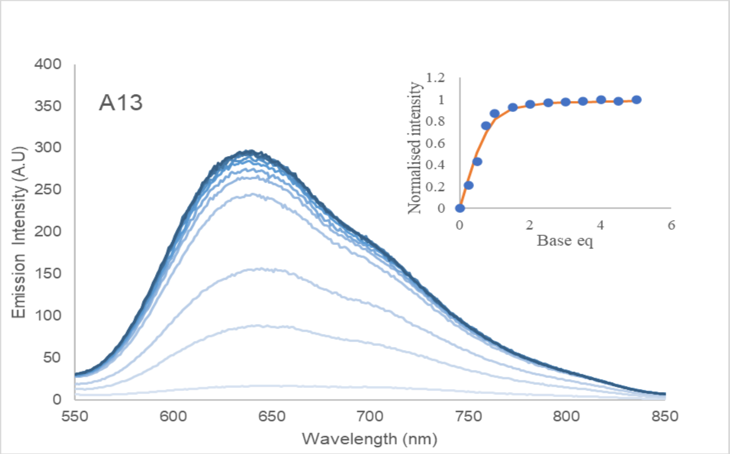

Figure S18: Change in emission of Ru-PDC3 (10 µM) with increasing concentration of A13 (0-50 µM) representative binding curve. Aqueous buffer solution is 10 mM potassium phosphate buffer with 100 mM KCl at pH7.

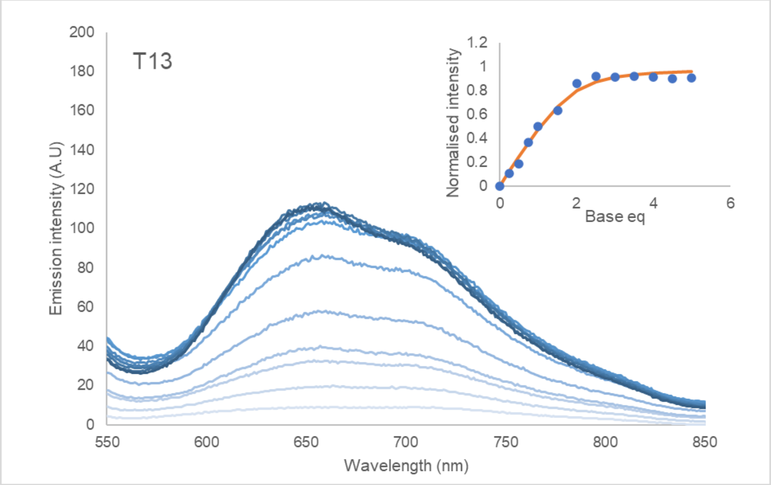

Figure S19: Change in emission of Ru-PDC3 (10 µM) with increasing concentration of T13 (0-50 µM) representative binding curve Aqueous buffer solution is 10 mM potassium phosphate buffer with 100 mM KCl at pH7.

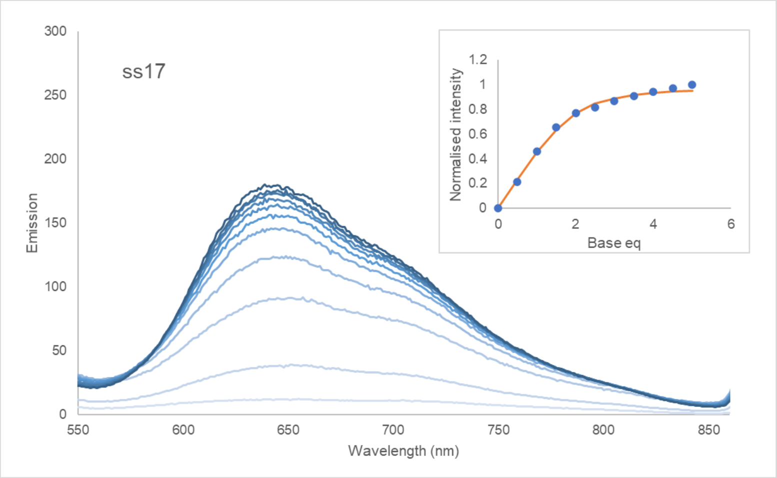

Figure S20: Change in emission of Ru-PDC3 (10 µM) with increasing concentration of ss17 (0-50 µM) representative binding curve Aqueous buffer solution is 10 mM potassium phosphate buffer with 100 mM KCl at pH7.

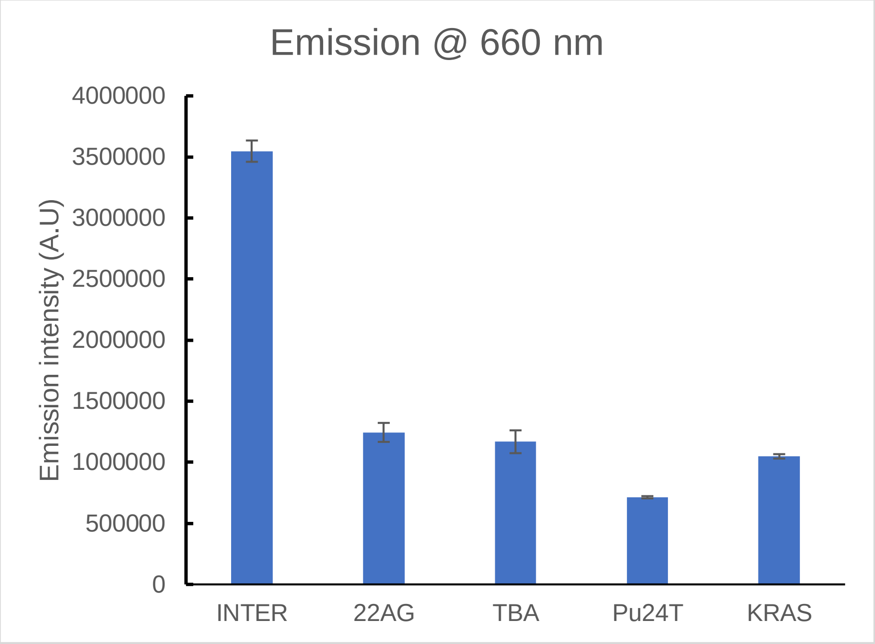

Figure S21: Emission intensity of Ru-PDC3 (10 µM) when bound to G4s at a ratio of [3]:[1], [G4]:[Ru] in KPi buffer. Excitation wavelength = 450 nm. The samples were absorbance matched at the excitation wavelength.

Table S2: Calculated binding constants, site sizes and sum of square residuals for each DNA titration and computationally calculated binding affinities based on molecular docking

Sequence	Secondary structure	**PDB**	**Binding constant K_b (M^{-1})**	**Site Size**	**Sum of squared residuals**
22 AG	Mixed hybrid	1kf1	$2.2 \pm 0.7 \times 10^7$	0.5	3.6%
KRAS	Parallel	5I2V	$1.4 \pm 0.1 \times 10^7$	0.5	3.8%
Pu24t	Parallel	2MGN	$2.6 \pm 0.1 \times 10^6$	0.5	3.4%
Inter	Intermolecular	-	$1.4 \pm 0.2 \times 10^7$	0.5	3.2%
TBA	Anti-parallel	148D	$5.6 \pm 1.7 \times 10^6$	0.5	4.9%
ctDNA	Double stranded	-	$2.3 \pm 0.6 \times 10^6$	1	3.7%
ss17	Single stranded	-	$1.3 \pm 0.4 \times 10^6$	1	1.4%
ds17	Double stranded	-	$1.9 \pm 0.3 \times 10^6$	1	2.2%
A13	Single stranded	-	$2.3 \pm 1.2 \times 10^6$	0.5	1.2%
t13	Single stranded	-	$1.4 \pm 0.6 \times 10^6$	1	1.9%

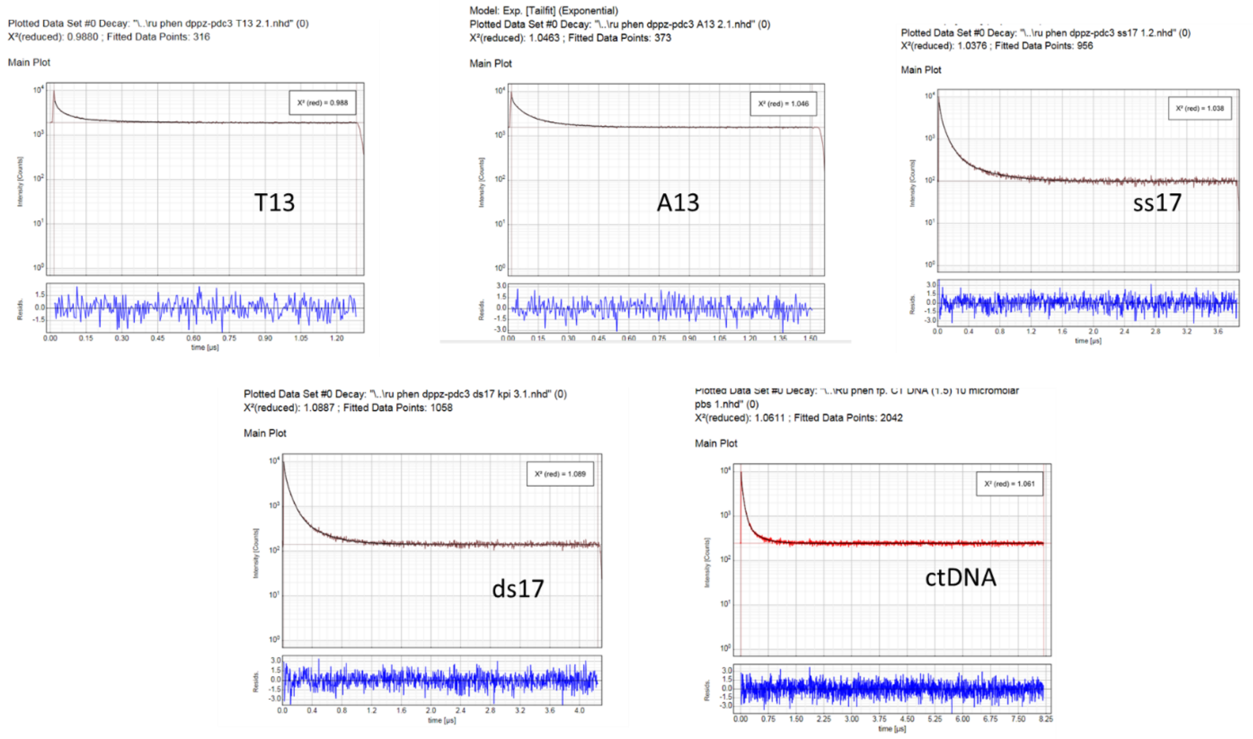

Figure S22: Lifetime decays of Ru-PDC (10 µM) with single stranded and double stranded DNA (50 µM)

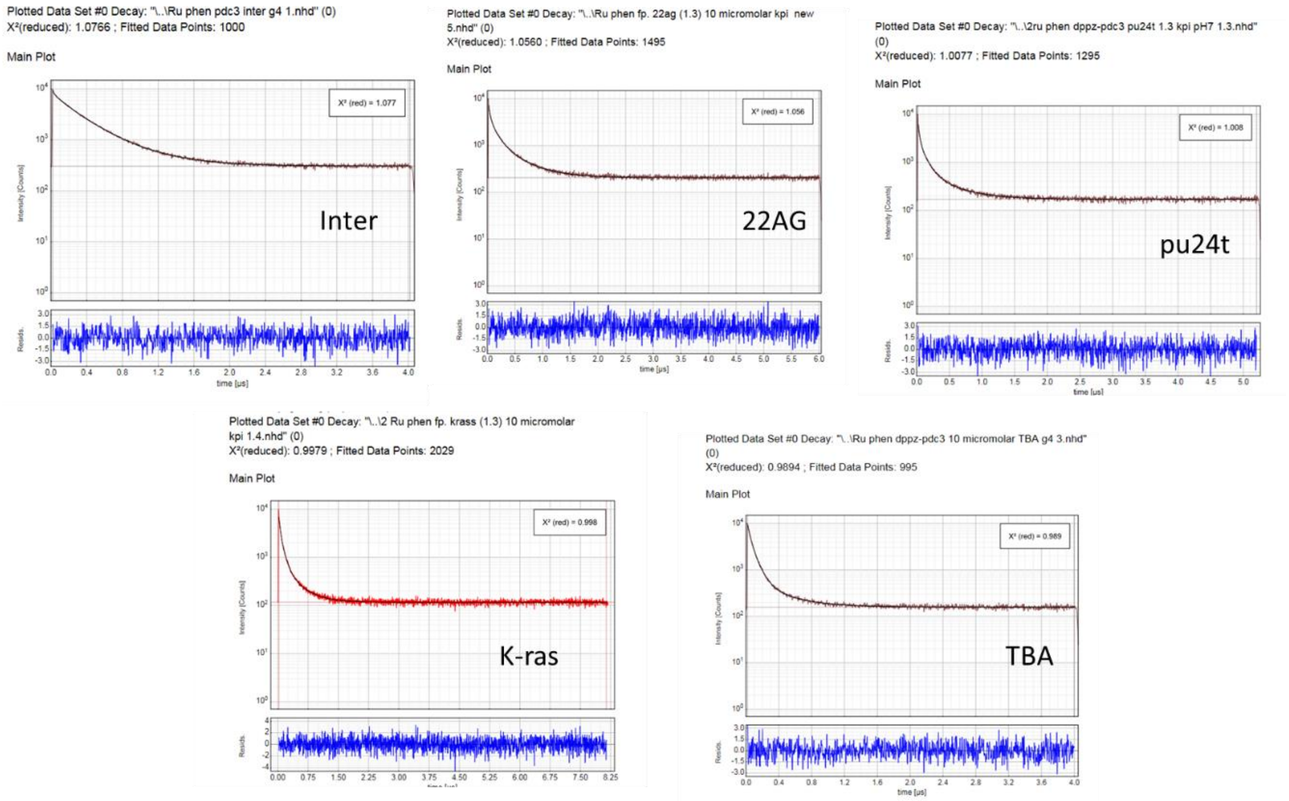

Figure S23: Lifetime decays of Ru-PDC (10 µM) with single stranded and double stranded DNA (30 µM).

Circular Dichroism

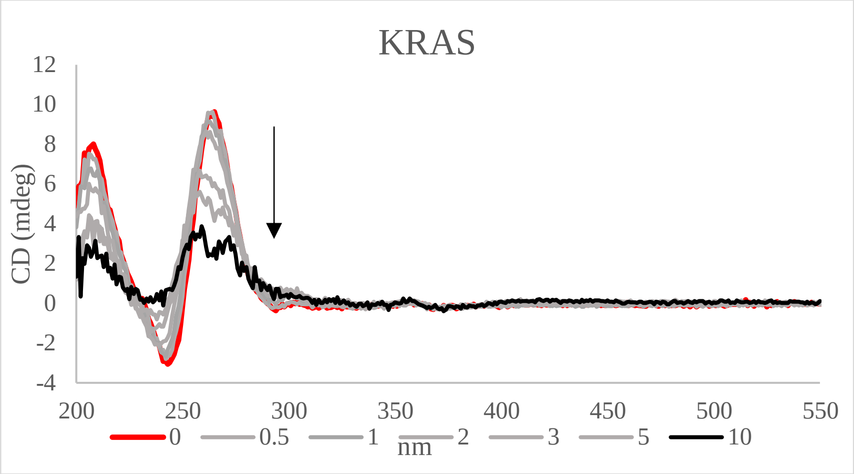

Figure S24: Structural changes to a circular dichroism spectra of KRAS (5 µM) upon addition of Ru-PDC3 (0-50 µM) in Kpi buffer. The arrow indicates the decrease in ellipticity upon titration with Ru-PDC3.

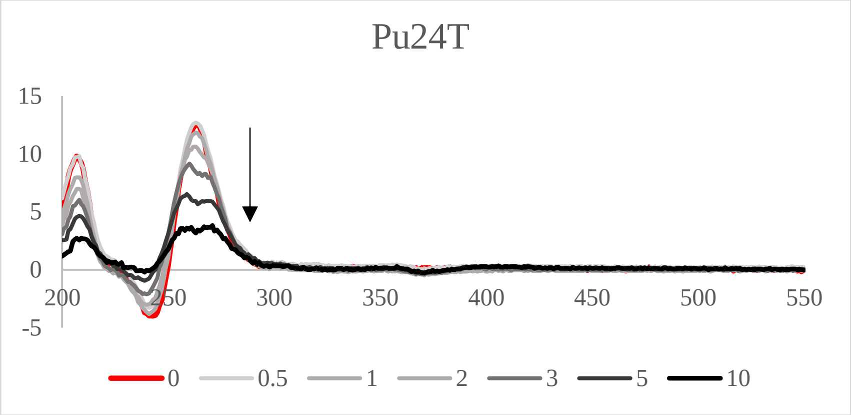

Figure S25: Structural changes to circular dichroism spectra of Pu24T (5 µM) upon addition of Ru-PDC3 (0-50 µM) in Kpi buffer. The arrow indicates the decrease in ellipticity upon titration with Ru-PDC3.

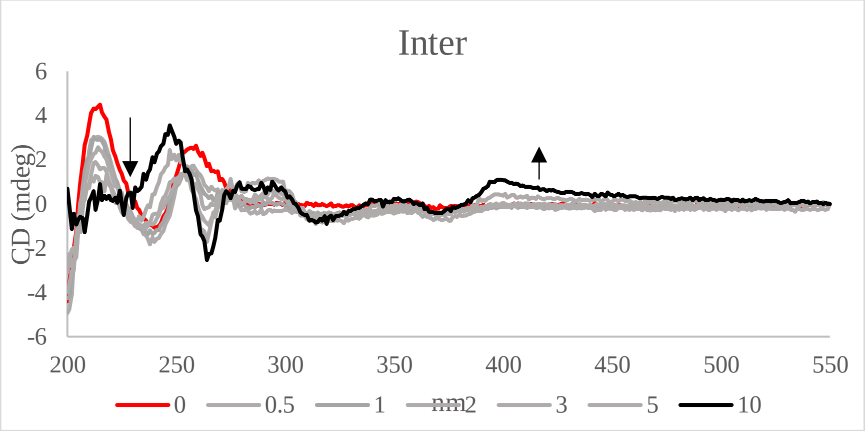

Figure S26: Structural changes to circular dichroism spectra of inter (5 µM) upon addition of Ru-PDC3 (0-50 µM) in Kpi buffer. The arrows indicate the decrease in ellipticity upon titration with Ru-PDC3 and the emergence of a ICD.

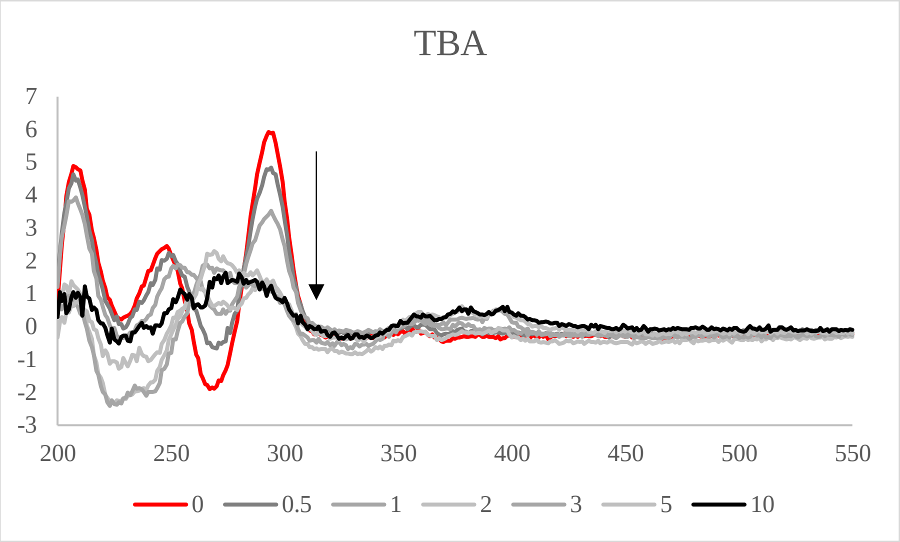

Figure S27: Structural changes to circular dichroism spectra of TBA (5 µM) upon addition of Ru-PDC3 (0-50 µM) in Kpi buffer. The arrow indicates the decrease in ellipticity upon titration with Ru-PDC3.

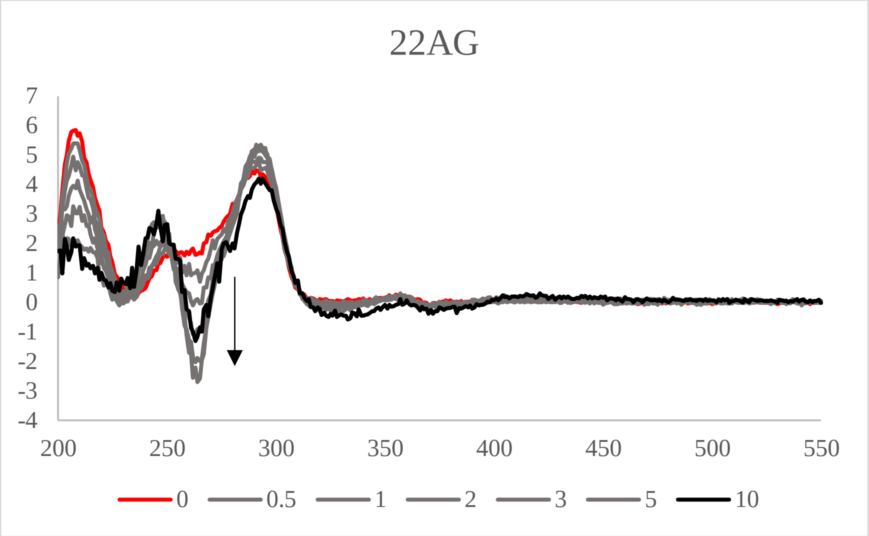

Figure S28: Structural changes to circular dichroism spectra of TBA (5 µM) upon addition of Ru-PDC3 (0-50 µM) in kpi buffer. The arrow indicates the decrease in ellipticity upon titration with Ru-PDC3.

Table S3: Variation CD signal for each quadruplex and the wavelength at which observations are made.

	structure	(nm)	0.5	1	2	3	5	10
INTER	Intermolecular	210	-31	-34	-42	-61	-72	-95
Pu24T	Parallel	265	1	-7	-18	-32	-53	-73
KRAS	Parallel	265	-3	-8	-16	-37	-57	-71
TBA	Anti-parallel	295	-21	-43	-76	-82	-79	-82
22AG	Mixed hybrid	295	7	12	17	23	19	-5

Raman Studies

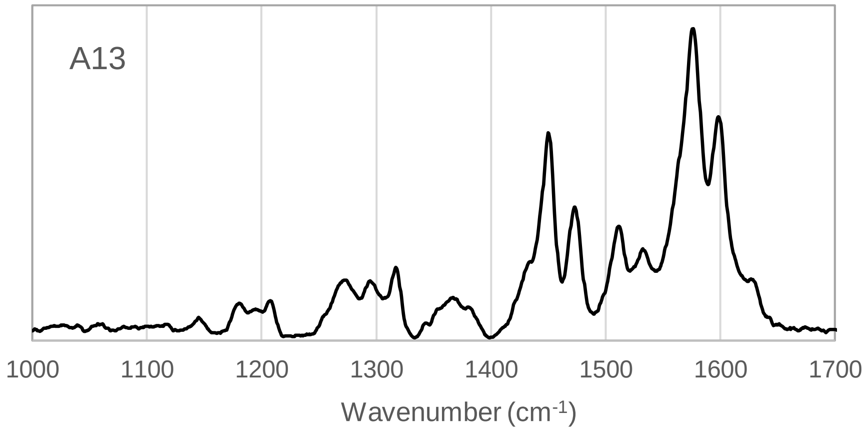

Figure S29: Ru-PDC3 (100 µM) bound to A13 (500 µM) in KPi buffer pH 7.4 (λ=473 nm)

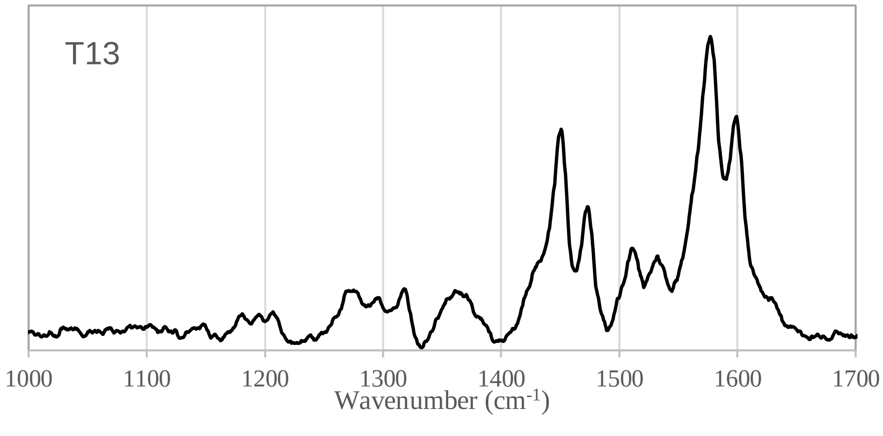

Figure S30: Ru-PDC3 (100 µM) bound to T13 (500 µM) in KPi buffer pH 7.4 (λ=473 nm)

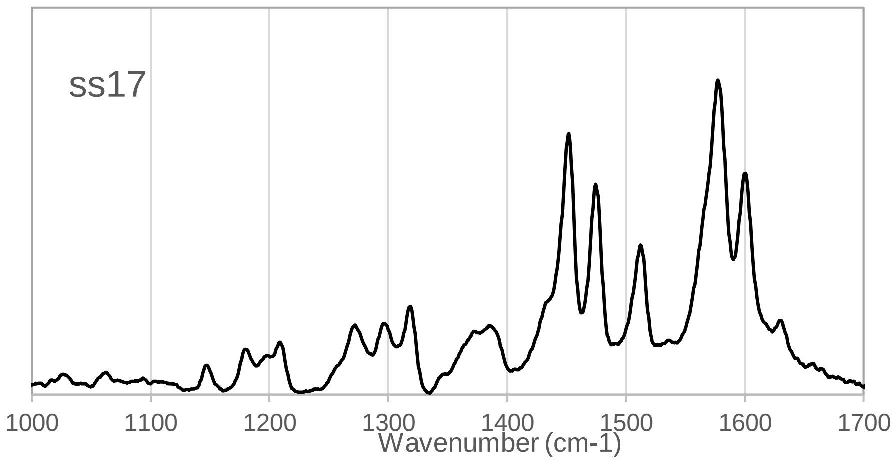

Figure S31: Ru-PDC3 (100 µM) bound to ss17 (500 µM) in KPi buffer pH 7.4 (λ=473 nm)

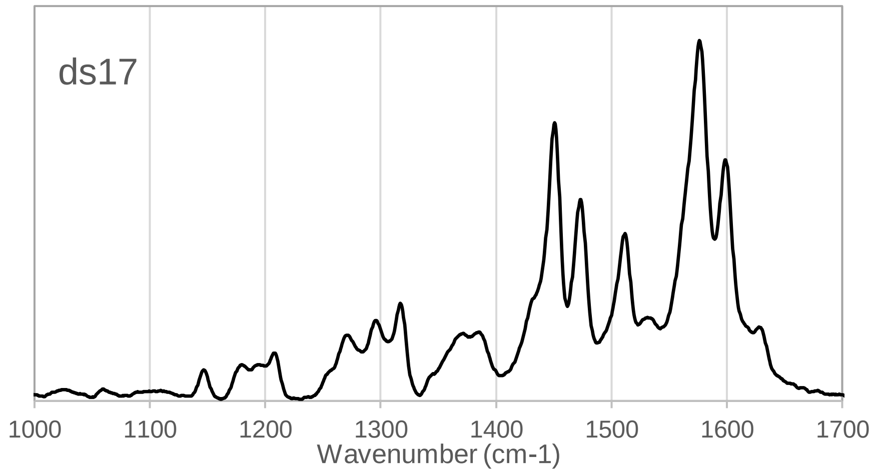

Figure S32: Ru-PDC3 (100 µM) bound to ds17 (500 µM) in KPi buffer pH 7.4 (λ=473 nm)

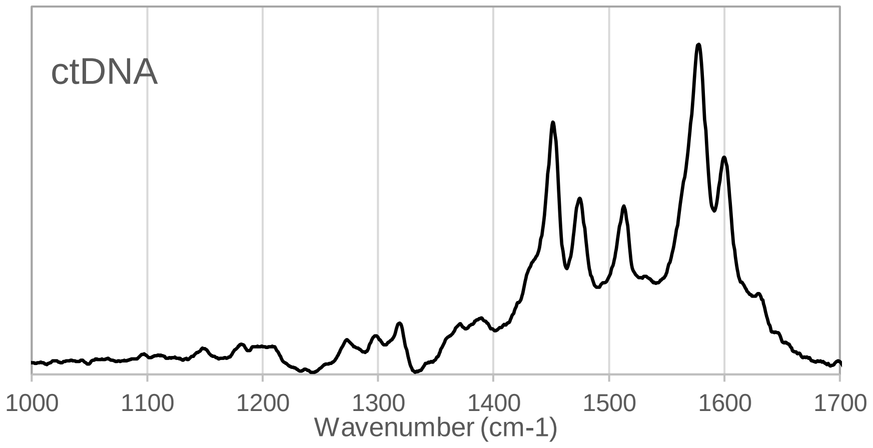

Figure S33: Ru-PDC3 (100 µM) bound to ctDNA (500 µM) in KPi buffer pH 7.4 (λ=473 nm)

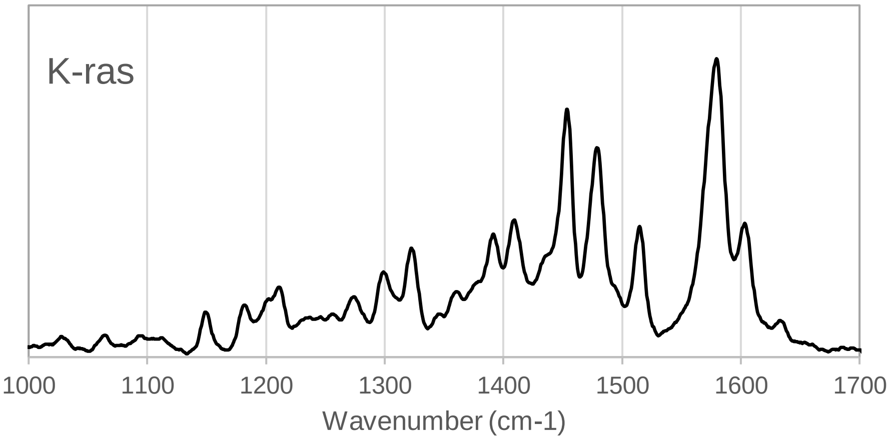

Figure S34: Resonance Raman spectroscopy Ru-PDC3 (100 µM) bound to KRAS 300 µM in KPi buffer pH 7.4 (λ=473 nm)

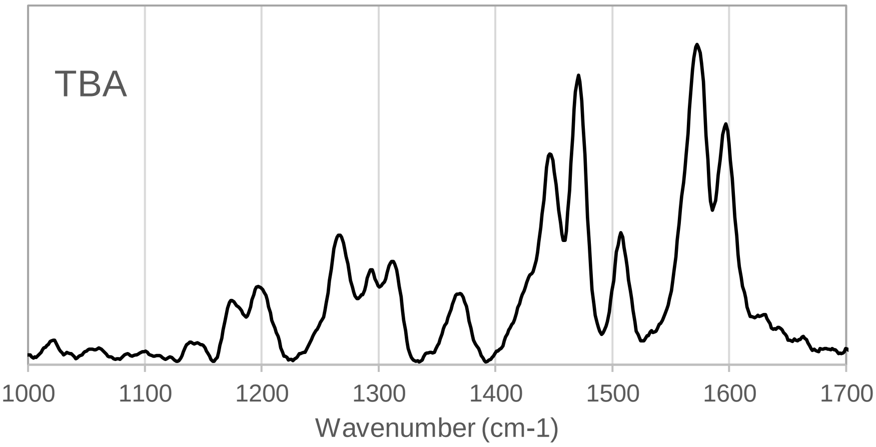

Figure S35: Ru-PDC3 (100 µM) bound to TBA (300 µM) in KPi buffer pH 7.4 (λ=473 nm)

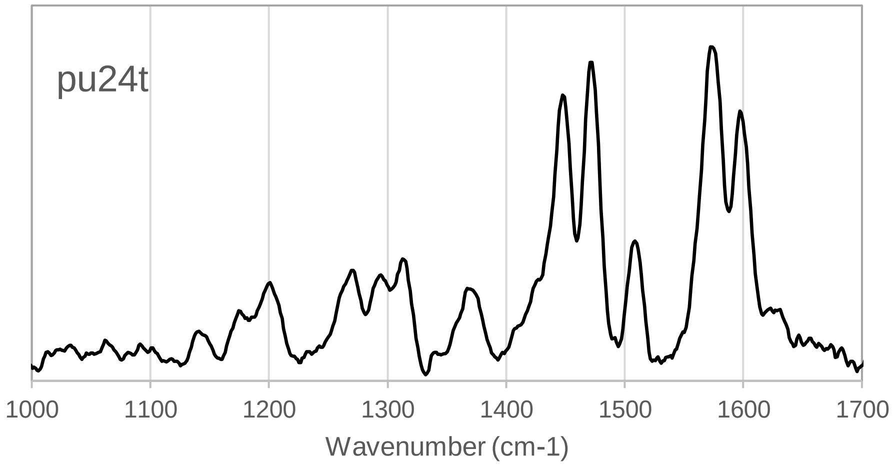

Figure S36: Ru-PDC3 (100 µM) bound to pu24T (300 µM) in KPi buffer pH 7.4 (λ=473 nm)

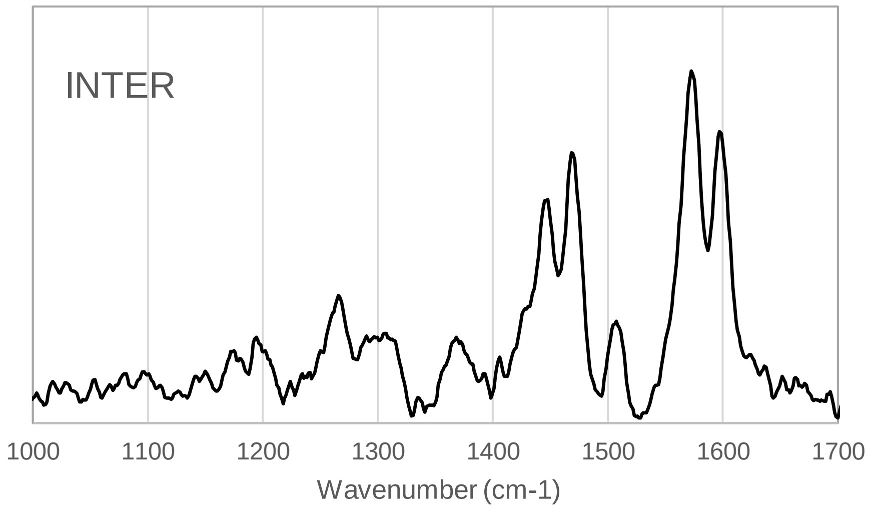

Figure S37: Ru-PDC3 (100 µM) bound to INTER (300 µM) in KPi buffer pH 7.4 (λ=473 nm)

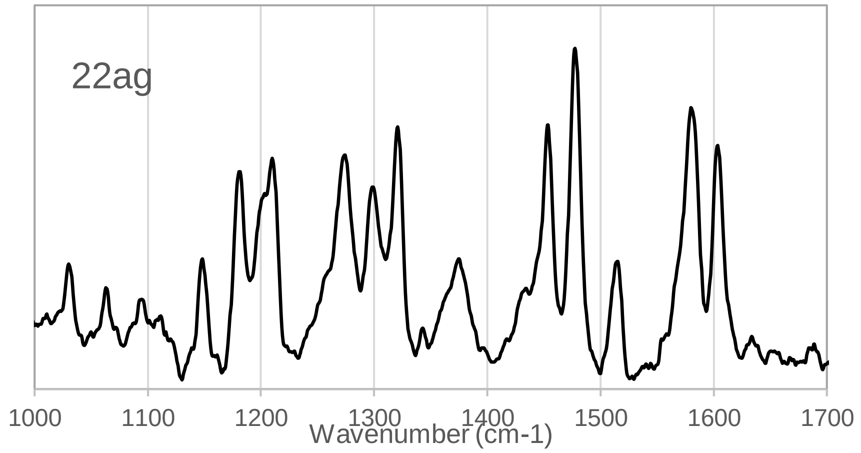

Figure S38: Ru-PDC3 (100 µM) bound to 22AG (300 µM) in KPi buffer pH 7.4 (λ=473 nm)

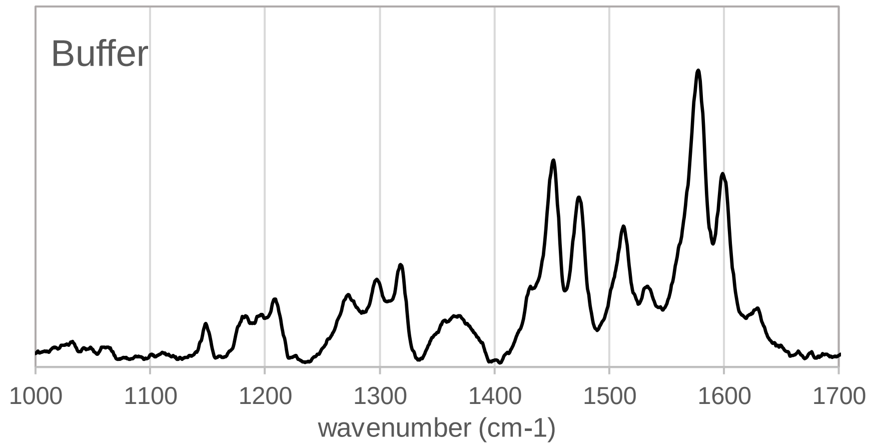

Figure S39: Ru-PDC3 (100 µM) KPi buffer pH 7.4 (λ=473 nm)

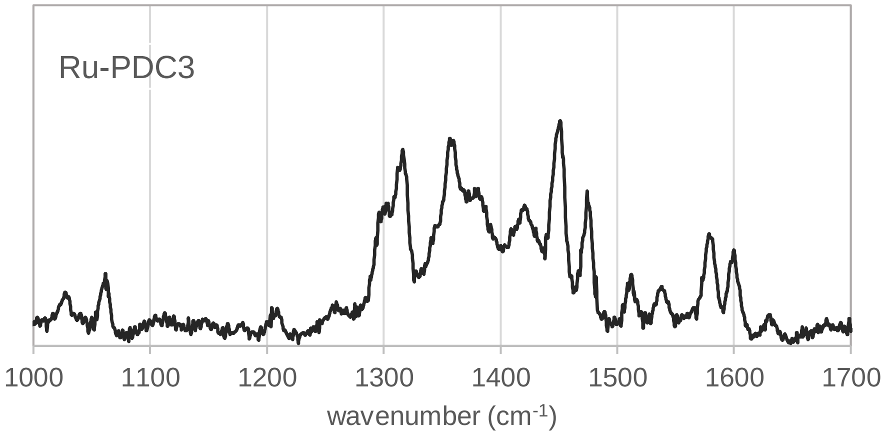

Figure S40: Ru-PDC3(100 µM) bound to A13 µ500 M in KPi buffer pH 7.4 (λ=785 nm)

Table S4: Estimates of Free Energy of binding of enantiomers of RuPDC3 from molecular docking simulations with selected G4 Quadruplexes.

	22AG		KRAS	
	LPhen2	DPhen2	LPhen2	DPhen2
Estimated Free kCal/mol	-10.29	-10.86	-10.78	-10.17

Cell studies

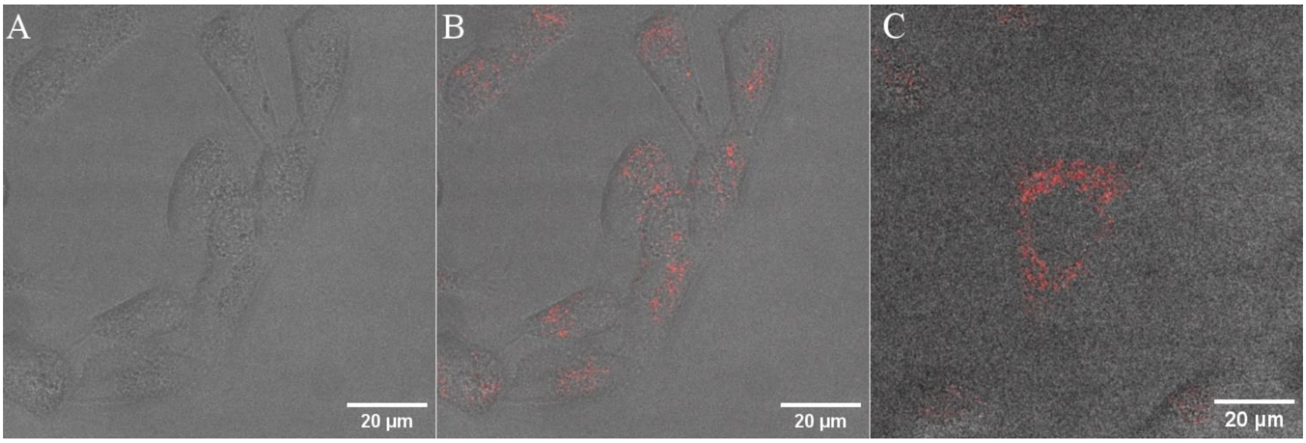

Figure S41: Confocal images of A549 cells treated with $[Ru(phen)_2(tpphz\text{-}PDC3)]^{4+}$ at 50 µM/ 24 h. Shown are the A) Bright-field only and the B, C) Ru(II) channel/ brightfield overlay images. A 475 nm white light laser was used to excite the ruthenium complex and emission was collected between 550 and 800 nm.

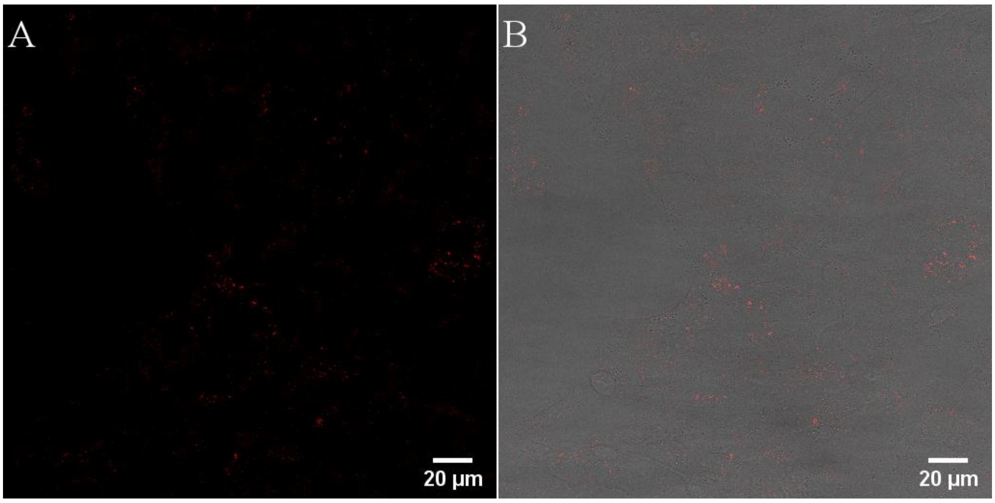

Figure S42: Confocal images of live A549 cells pre-treated with oligomycin (5 µM) and 2-Deoxy-D-Glucose (50 mM) for 40 minutes prior to incubation with $[Ru(phen)_2(tpphz\text{-}PDC3)]^{4+}$ at 50 µM for 24 h. Shown is the A) Ru(II) channel and the B) Ru(II) channel/brightfield overlay image. The image is acquired at a position focusing on the exterior cell membrane. A 475 nm white light laser was used to excite the ruthenium complex and emission was collected between 550 and 800 nm.

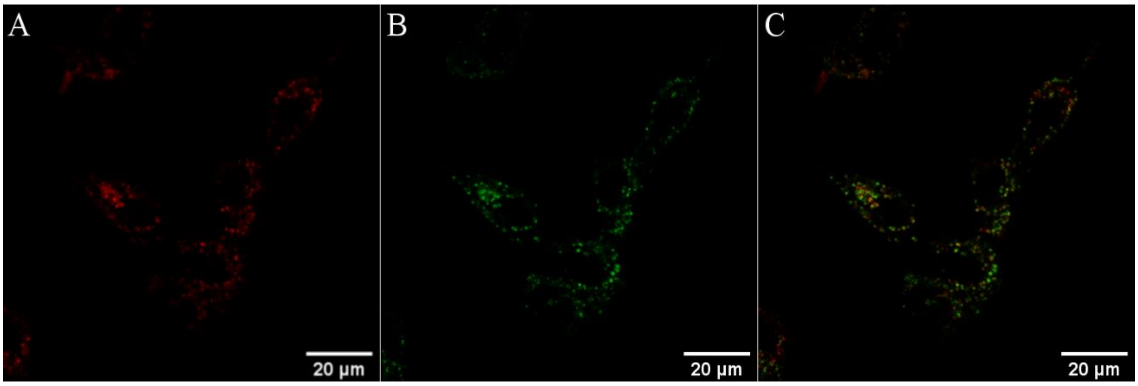

Figure S43: Representative confocal images of live A549 cells treated with A) $[Ru(phen)_2(tpphz\text{-}PDC3)]^{4+}$ at **50 µM** for **24 h** and co-stained with B) LysoTracker Green DND 26 (60 nM); C) overlay of channels (Pearson's coefficient, r = 0.51 ± 0.03). A 475 nm white light laser was used to excite the ruthenium complex and emission was collected between 550 and 800 nm. LysoTracker Green DND 26 was excited at 504 nm and emission was collected between 510 – 515 nm.

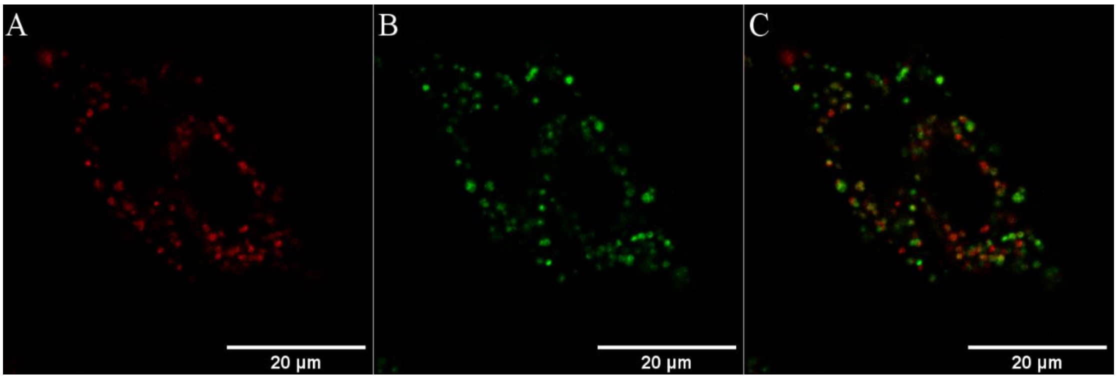

Figure S44: Representative confocal images of live A549 cells treated with $[Ru(phen)_2(tpphz\text{-}PDC3)]^{4+}$ at **50 µM** for **48 h** and co-stained with LysoTracker Green DND 26 (60 nM). A)

[Ru(phen)$_2$(tpphz-PDC3)]$^{4+}$ channel B) LysoTracker Green DND 26 channel and C) overlay of channels (Pearson's coefficient, r = 0.56 ± 0.01). A 475 nm white light laser was used to excite the ruthenium complex and emission was collected between 550 and 800 nm. LysoTracker Green DND 26 was excited at 504 nm and emission was collected between 510 – 515 nm.

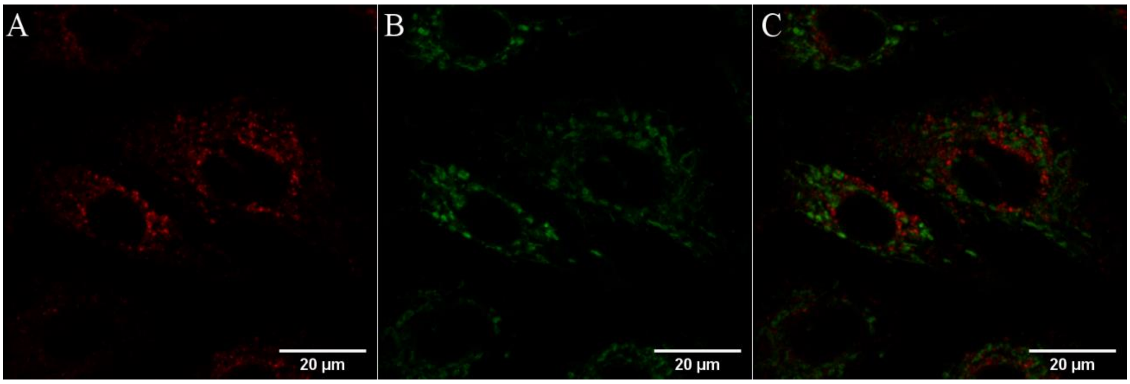

Figure S45: Confocal images of live A549 cells treated with A) [Ru(phen)$_2$(tpphz-PDC3)]$^{4+}$ at **50 µM** for **48 h** and co-stained with B) BioTracker 488 Green (100 nM); C) overlay channels. A 475 nm white light laser was used to excite the ruthenium complex and emission was collected between 550 and 800 nm. BioTracker 488 Green (100 nM) was excited at 488 nm and emission was collected between 510 – 520 nm.

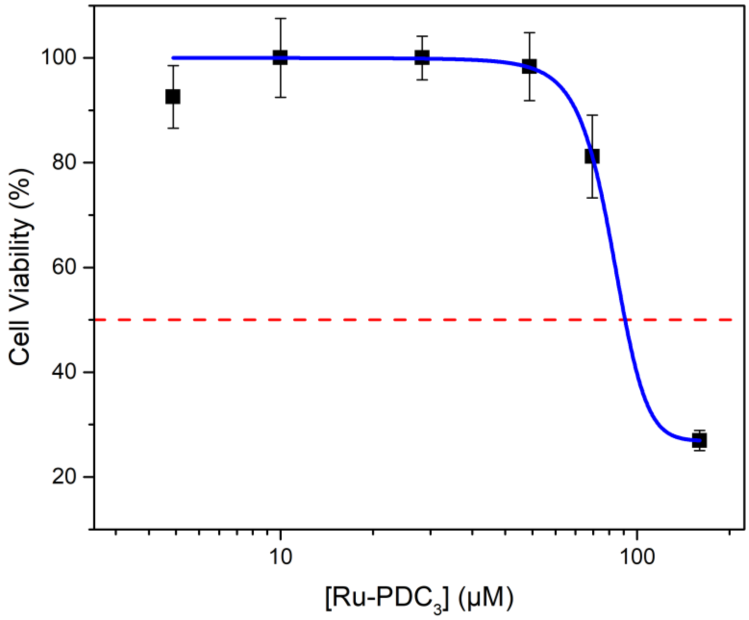

Figure S46: Dose-response curve for [Ru(phen)$_2$(tpphz-PDC3)]$^{4+}$. Live A549 cells were treated with the complex for 24 h followed by addition of Resazurin for 6 h (n=3). Absorbance readings were carried out at 570 nm and 600 nm (corrected for background subtraction). Data were expressed as averaged percentages compared to non-treated cells.

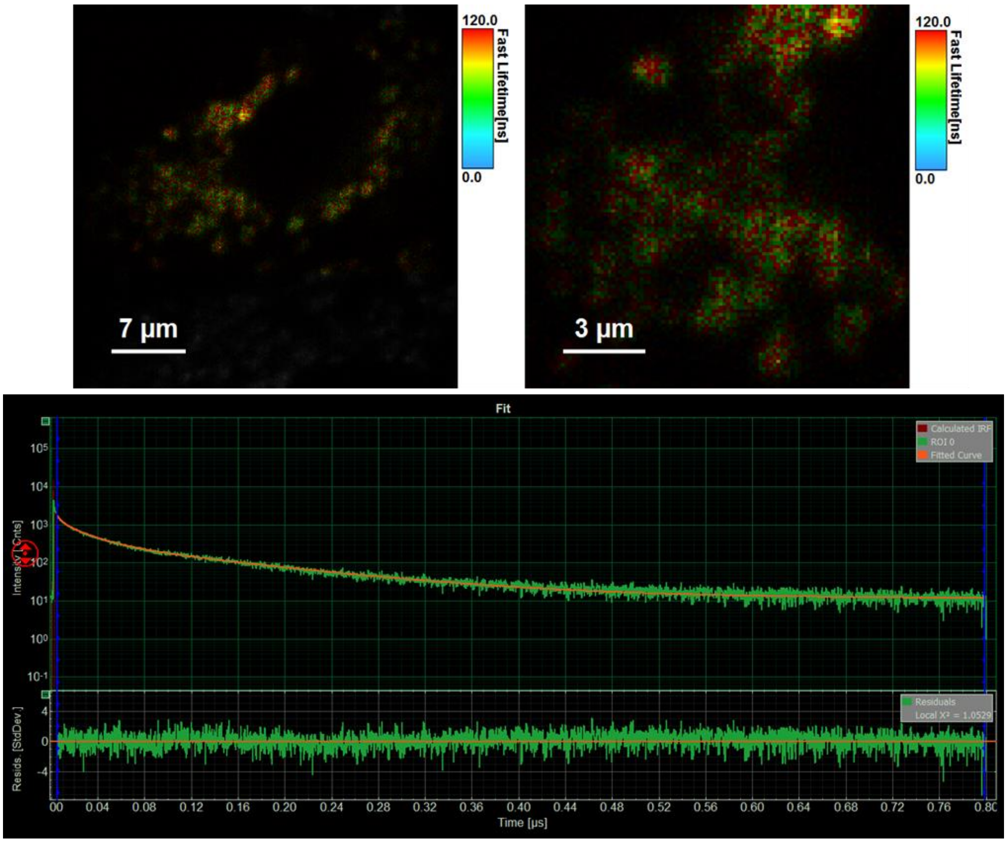

Figure S47: Luminescent lifetime image and corresponding decay [Ru(phen)$_2$(tpphz-PDC3)]$^{4+}$ in a single live A549 cell. The decay was fitted to a tri-exponential model with lifetimes of 114.1 ± 1.3 ns, 22.73 ± 0.72 ns and 3.99 ± 0.34 ns.

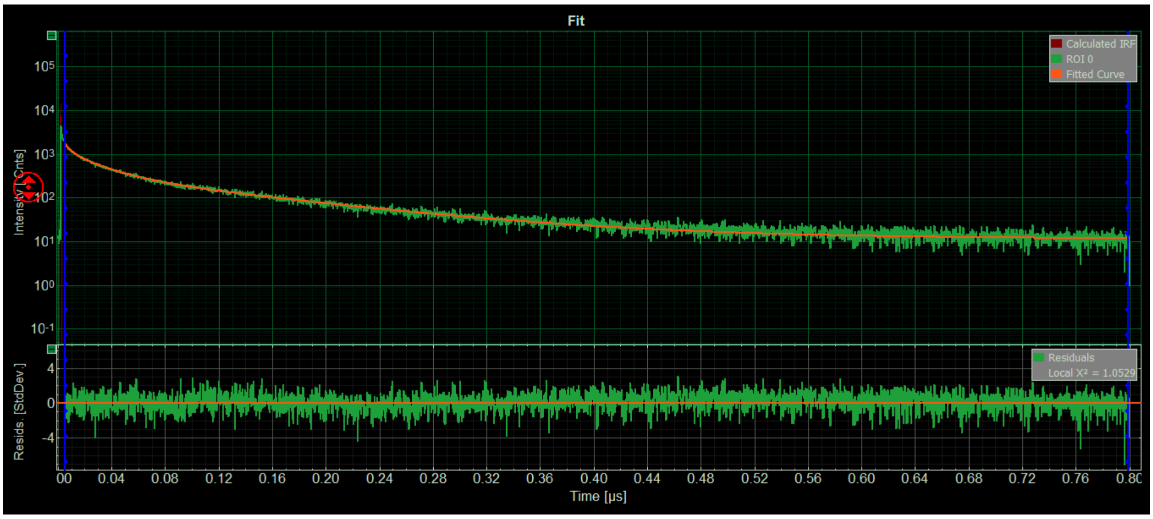

Figure S48: The PLIM decay for the region of interest (ROI) selected (single A549 cell) was fitted to a tri-exponential model with lifetimes of 120.4 ± 3.7 ns, 22.7 ± 1.2 ns and 3.17 ± 0.14 ns (χ^2 = 1.0360). The PLIM image was acquired by exciting at 405 nm and emission collected between 550 and 800nm.

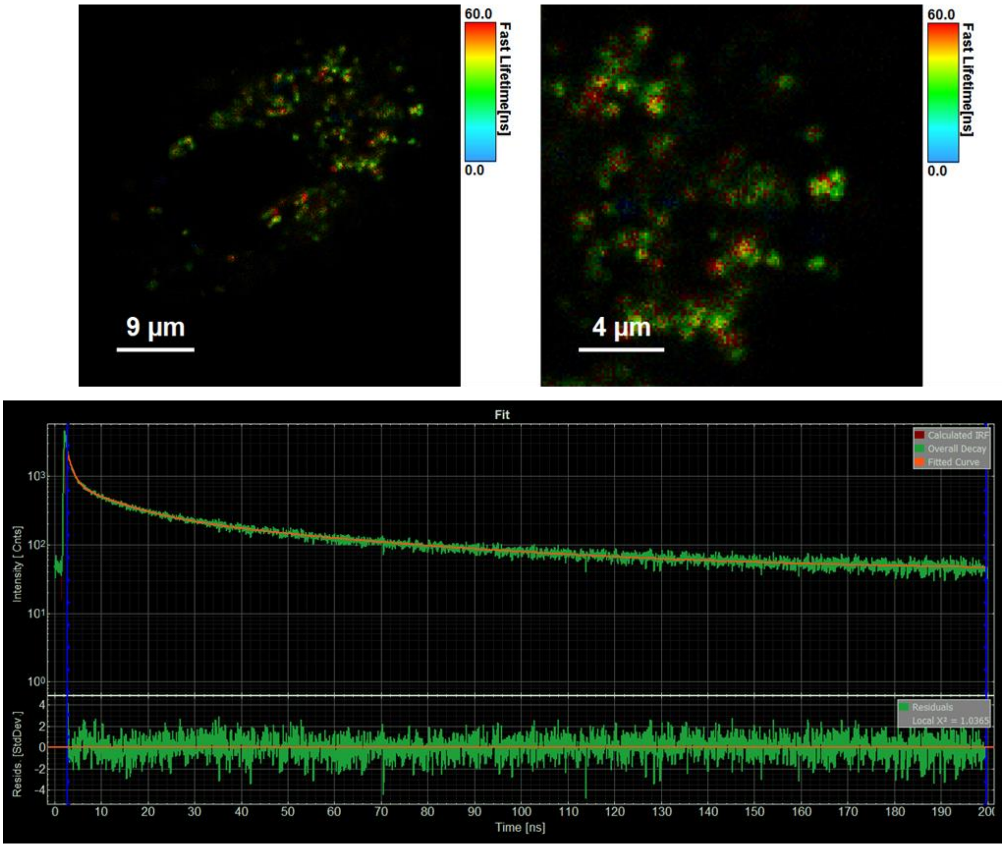

Figure S49: Representative luminescent lifetime imaging microscopy and fits for $[Ru(phen)_2(tpphz\text{-}PDC3)]^{4+}$ (50 µM / 24 h) in a single live A549 cell. The decay was fitted to a tri-exponential model with lifetimes 50.5 ± 1.8 ns, 9.51 ± 0.57 ns and 1.10 ± 0.06 ns.

References

(1) Marchand, A.; Gabelica, V. Folding and Misfolding Pathways of G-Quadruplex DNA. *Nucleic Acids Res* **2016**, *44* (22), 10999–11012. https://doi.org/10.1093/nar/gkw970.

(2) D'Aria, F.; D'Amore, V. M.; Di Leva, F. S.; Amato, J.; Caterino, M.; Russomanno, P.; Salerno, S.; Barresi, E.; De Leo, M.; Marini, A. M.; Taliani, S.; Da Settimo, F.; Salgado, G. F.; Pompili, L.; Zizza, P.; Shirasawa, S.; Novellino, E.; Biroccio, A.; Marinelli, L.; Giancola, C. Targeting the KRAS Oncogene: Synthesis, Physicochemical and Biological Evaluation of Novel G-Quadruplex DNA Binders. *European Journal of Pharmaceutical Sciences* **2020**, *149*, 105337. https://doi.org/10.1016/j.ejps.2020.105337.

(3) Reshetnikov, R. V.; Sponer, J.; Rassokhina, O. I.; Kopylov, A. M.; Tsvetkov, P. O.; Makarov, A. A.; Golovin, A. V. Cation Binding to 15-TBA Quadruplex DNA Is a Multiple-Pathway Cation-Dependent Process. *Nucleic Acids Res* **2011**, *39* (22), 9789–9802. https://doi.org/10.1093/nar/gkr639.

(4) McQuaid, K.; P. Hall, J.; Baumgaertner, L.; J. Cardin, D.; J. Cardin, C. Three Thymine/Adenine Binding Modes of the Ruthenium Complex Λ-[Ru(TAP) 2 (Dppz)] 2+ to the G-Quadruplex Forming Sequence d(TAGGGTT) Shown by X-Ray Crystallography. *Chemical Communications* **2019**, *55* (62), 9116–9119. https://doi.org/10.1039/C9CC04316K.

(5) Renard, I.; Grandmougin, M.; Roux, A.; Yang, S. Y.; Lejault, P.; Pirrotta, M.; Wong, J. M. Y.; Monchaud, D. Small-Molecule Affinity Capture of DNA/RNA Quadruplexes and Their Identification in Vitro and in Vivo through the G4RP Protocol. *Nucleic Acids Res* **2019**, *47* (11), 5502–5510. https://doi.org/10.1093/nar/gkz215.
